# Genome-wide sequencing of small RNAs reveals a tissue-specific loss of conserved microRNA families in *Echinococcus granulosus*

**DOI:** 10.1186/1471-2164-15-736

**Published:** 2014-08-29

**Authors:** Yun Bai, Zhuangzhi Zhang, Lei Jin, Hui Kang, Yongqiang Zhu, Lu Zhang, Xia Li, Fengshou Ma, Li Zhao, Baoxin Shi, Jun Li, Donald P McManus, Wenbao Zhang, Shengyue Wang

**Affiliations:** Shanghai-MOST Key Laboratory of Health and Disease Genomics, Chinese National Human Genome Center at Shanghai, 250 Bibo Road, Shanghai, 201203 China; Veterinary Research Institute, Xinjiang Academy of Animal Sciences, 151 East-Kelamayi Street, Urumqi, Xinjiang 830000 China; State Key Laboratory Incubation Base of Xinjiang Major Diseases Research, Clinical Medical Research Institute, The First Affiliated Hospital of Xinjiang Medical University, No. 1 Liyushan Road, Urumqi, Xinjiang 830054 China; Molecular Parasitology Laboratory, QIMR Berghofer Institute of Medical Research, Brisbane, QLD Australia

**Keywords:** *Echinococcus granulosus*, microRNA, Deep sequencing, Differential expression, Life cycle stage development

## Abstract

**Background:**

MicroRNAs (miRNAs) are important post-transcriptional regulators which control growth and development in eukaryotes. The cestode *Echinococcus granulosus* has a complex life-cycle involving different development stages but the mechanisms underpinning this development, including the involvement of miRNAs, remain unknown.

**Results:**

Using Illumina next generation sequencing technology, we sequenced at the genome-wide level three small RNA populations from the adult, protoscolex and cyst membrane of *E. granulosus*. A total of 94 pre-miRNA candidates (coding 91 mature miRNAs and 39 miRNA stars) were *in silico* predicted. Through comparison of expression profiles, we found 42 mature miRNAs and 23 miRNA stars expressed with different patterns in the three life stages examined. Furthermore, considering both the previously reported and newly predicted miRNAs, 25 conserved miRNAs families were identified in the *E. granulosus* genome. Comparing the presence or absence of these miRNA families with the free-living *Schmidtea mediterranea*, we found 13 conserved miRNAs are lost in *E. granulosus*, most of which are tissue-specific and involved in the development of ciliated cells, the gut and sensory organs. Finally, GO enrichment analysis of the differentially expressed miRNAs and their potential targets indicated that they may be involved in bi-directional development, nutrient metabolism and nervous system development in *E. granulosus*.

**Conclusions:**

This study has, for the first time, provided a comprehensive description of the different expression patterns of miRNAs in three distinct life cycle stages of *E. granulosus*. The analysis supports earlier suggestions that the loss of miRNAs in the Platyhelminths might be related to morphological simplification. These results may help in the exploration of the mechanism of interaction between this parasitic worm and its definitive and intermediate hosts, providing information that can be used to develop new interventions and therapeutics for the control of cystic echinococcosis.

**Electronic supplementary material:**

The online version of this article (doi:10.1186/1471-2164-15-736) contains supplementary material, which is available to authorized users.

## Background

MicroRNAs (miRNAs) are a class of endogenous non-coding RNAs of around 22 nucleotides (nts) in length. They play a role as post-transcriptional regulators, partially or completely complementary binding to messenger RNA transcripts (mRNAs), usually resulting in direct degradation or translational repression of target genes [1, 2]. In eukaryotes, miRNAs are involved in a broad variety of biological processes [3], such as embryonic development, cell proliferation, cell differentiation, and apoptosis. To date, more than twenty thousand miRNAs from 223 species have been registered in the miRBase (http://www.mirbase.org/, release 21.0, June 2014) [4]. miRNAs are usually highly conserved throughout the animal kingdom [2]. They have been continuously added to Metazoan genomes. The emergence of vertebrates is characterized by a strong increase in miRNA families, and correlates with the increase in vertebrate morphological complexity [5–7]. Therefore, miRNAs may have significantly contributed to phenotypic evolution in animals. Recently, a high rate of the loss of conserved miRNA loci has been found in the nematodes and the flatworms [8, 9]. This result suggested that the miRNA losses in Platyhelminths are not random events. However, to date, there is no report about the mechanisms of miRNA losses in metazoan phylogenetics.

*Echinococcus granulosus* is a flatworm and member of the Platyhelminthes, and is the causative agent of cystic echinococcosis (CE), a disease that is distributed throughout most areas of the world [10, 11]. Currently, up to 3 million people are infected with *E. granulosus* [12], and, in some areas, 10% of the population has detectable hydatid cysts by abdominal ultrasound and chest X-ray [13, 14]. This tapeworm requires two mammalian hosts to complete its life cycle. The mature adult worm resides in the small intestine of a carnivore (definitive host) and releases worm segments or proglottides containing hundreds of eggs which contaminate vegetation and water. When the eggs are swallowed by an intermediate host such as a sheep, the eggs hatch to release larval oncospheres into the digestive tract which are activated by bile and gastrointestinal enzymes. The activated oncospheres penetrate the intestinal wall and migrate via the circulatory system to various organs (mainly the liver and lungs). In these organs, the oncospheres develop into hydatid cysts over many months and the cysts generate brood capsules within which protoscoleces (PSC) are produced asexually. The cycle is completed when a canine (dog, wolf, fox) definitive host swallows PSC present in the hydatid cysts in infected offal, and the PSC develop into adult worms in the small intestine [15].

Although the life cycle of *E. granulosus* is well known, the mechanisms underlying the main developmental events throughout remain largely unclear. Our previous study of the transcriptome of *E. granulosus* revealed that 1,452 genes were up- or down-regulated in adult, PSC and cyst stages [16]. Moreover, a global proteomic analysis of the expression characteristics of *E. granulosus* in larval and adult stages identified 22 adult-specific and 263 PSC-specific proteins [17]. These studies suggested that transcriptional regulatory mechanisms are pivotal in the control of *E. granulosus* development.

In regards to flatworms, miRNAs have been experimentally identified in *Schmidtea mediterranea* [18, 19], *Schistosoma japonicum* [20–22] and *Schistosoma mansoni* [23, 24]. A PCR-based cloning study identified 26 mature miRNAs in PSC and the cystic stage of *E. granulosus* [25]. However, due to the hitherto restricted experimental methods and the limited genomic information available, the numbers and expression profile of *E. granulosus* miRNAs are still unclear. Here, we used next generation sequencing technology (NGS) to further explore the diversity of *E. granulosus* miRNAs and their expression patterns in different life stages. We expand the miRNA repertoire of *E. granulosus* and identify new miRNA encoding loci. Through comparing miRNA families in the Platyhelminths, we found that the losses of *E. granulosus* miRNAs may be associated with the loss of ciliated cells, the gut and sensory organs. The results significantly enhance our knowledge of miRNA species in *E. granulosus* and provide insights into miRNA evolution, biogenesis, and expression in parasites generally.

## Results

### Deep sequencing of three small RNA libraries from *E. granulosus*

**Figure 1:**
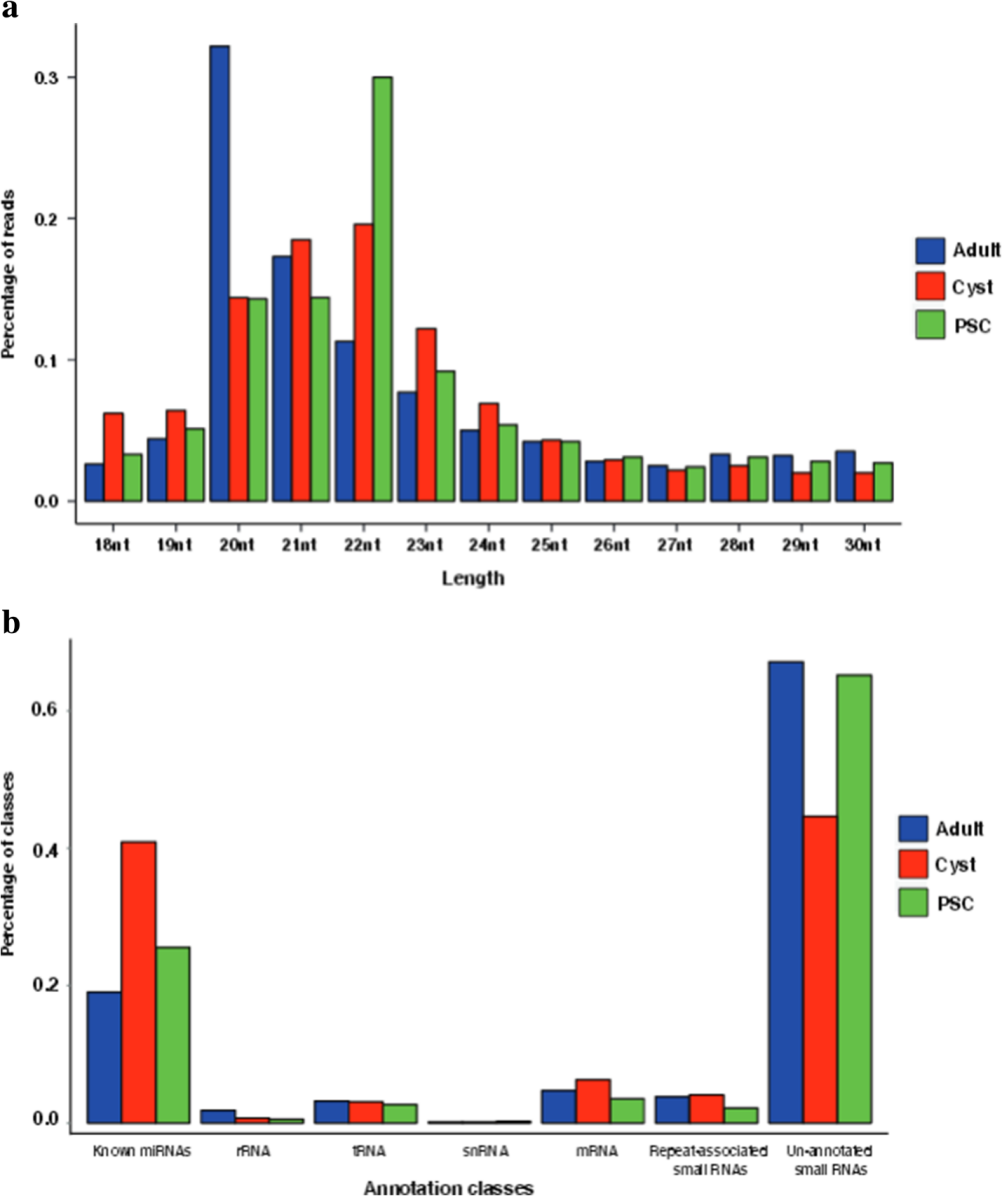
**Size distribution and classification of the small RNAs in the different libraries. (a)** Length distribution of the sequencing reads in the three libraries. The length percentages were calculated by dividing the counts of 18–30 nts reads in each library. The lengths of the small RNA reads are mainly distributed from 20 to 24 nts. **(b)** Classification of the sequenced small RNAs from adults, cyst and protoscoleces (PSC), respectively. The percentages were calculated by dividing the counts of reads matched to the genome.

To investigate the composition of small RNAs and the dynamic changes of miRNA expression during *E. granulosus* development, three small RNA libraries were constructed from adults, PSC and cyst membrane, and sequenced using Solexa sequencing technology. After removing low-quality sequences, adaptor contaminants and RNAs smaller than 18 nts, we obtained 23,632,021, 20,978,758 and 15,975,894 high-quality reads of small RNAs sized 18–30 nts from adults, PSC and cyst membrane, respectively [Additional file 1: Table S1]. Of these reads, 73.48% (adult), 73.31% (PSC) and 71.60% (cyst membrane) were 20 to 24 nts in length (Figure 1a), which is the typical size range for Dicer-derived products [26]. Through sequence mapping, 11,680,028, 12,966,593 and 9,375,095 reads from the three libraries perfectly matched to *E. granulosus* genome [16], [Additional file 1: Table S2]. After discarding known non-coding RNAs, such as rRNA, tRNA, snoRNA, repeat-associated RNA, and degraded fragments of mRNAs, the remaining 10,069,724, 11,775,532 and 8,025,262 small RNA reads from adults, PSC and cyst membrane, respectively, were used to search for both known and novel miRNAs (Figure 1b) [Additional file 1: Table S2].

### Identification of known and novel miRNAs from *E. granulosus*

To date, 23 mature miRNAs of *E. granulosus* have been identified [25] and are included in the miRBase database 20.0 (http://www.mirbase.org/). By deep sequencing, we found that all of the known mature miRNAs were present in our data sets [Additional file 1: Table S3], the majority being abundant in all three libraries. Furthermore, we also identified 23 miRNA stars from the known miRNA precursors [Additional file 1: Table S3].

In addition to known miRNAs, we also used miRDeep2 to predict and score novel miRNA precursors [27] and identified 94 miRNA candidates encoding 91 mature miRNAs and 39 miRNA stars [Additional file 1: Table S4 and S5]. All these miRNAs can be folded into characteristic miRNA stem-loop secondary hairpin structures and have a 1-2 nt 3′overhang pattern generated by Dicer cleavage during mature miRNA generation [Additional file 2]. We evaluated evolutionary conservations by homologous searches to known metazoan miRNAs and found 11 pre-miRNAs were classified into known families based on their precursor sequences, whereas 83 did not show homology with other miRNAs. We further matched these predicted pre-miRNA candidates to the *E. multilocularis* genome (http://www.sanger.ac.uk/cgi-bin/blast/submitblast/Echinococcus) and found 82 of 94 miRNA candidates were evolutionarily conserved (identity ≥87%) in both species [Additional file 1: Table S6]. To validate the novel miRNAs, we randomly selected 22 mature miRNAs and 5 miRNA stars, and conducted stem-loop RT-PCR [28]. All the selected miRNAs were expressed in *E. granulosus* [Additional file 3: Figure S1], suggesting that the filter criteria were sufficiently strict for predicting novel miRNAs.

**Figure 2:**
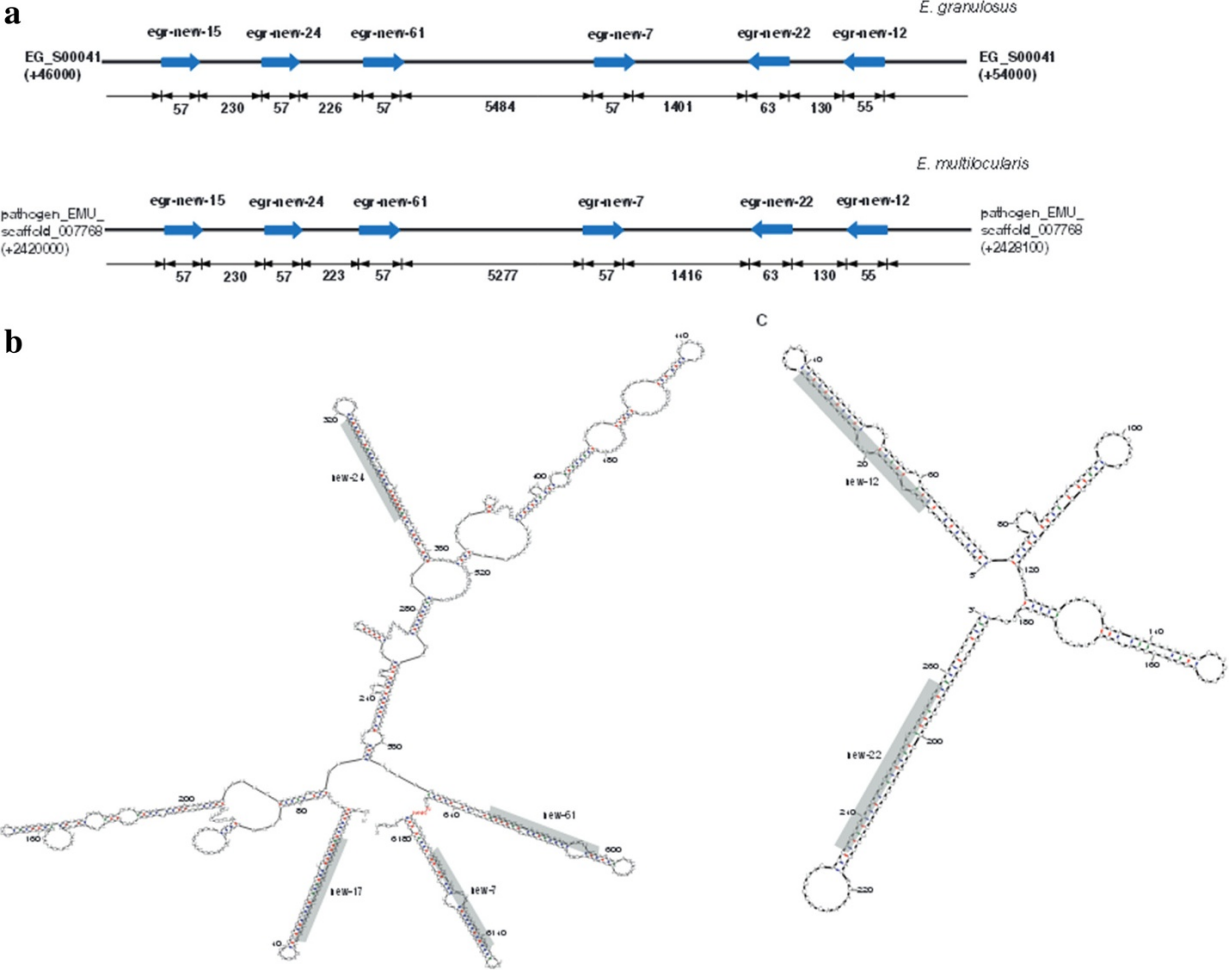
**Novel miRNAs and clusters detected in** ***E. granulosus.*** **(a)** The location of miRNA gene clusters in *E. granulosus* scaffolds. The rightward arrow indicates the location of the miRNA gene at the positive strand, while the leftward arrow indicates the miRNAs at the minus strand. The secondary structures of *Echinococcus* cluster containing new-17, new-24, new-61 and new-7 **(b)** and the cluster containing new-12 and new-22 **(c)** were predicted by mfold. The sequence of the mature miRNAs is shaded in grep. Limited by the size of the miRNA cluster, we used “N” instead of 5464 nt RNA sequences at the location between new-61 and new-7.

miRNA clusters are a group of miRNA genes located within a proximal distance on a chromosome [29]. In the present study, besides two published miRNA clusters, miR-71/2b/2d and miR-277/4988 [25], we identified two additional miRNA clusters located in closed loci (EG_S00041: 46,144-53,961 and pathogen_EMU_scaffold_007768: 2,420,386-2,428,006) in the genomes of both *E. granulosus* and *E. multilocularis* (Figure 2a). One cluster consists of four homologous novel miRNAs (new-15, new-24, new-61 and new-7) in the positive strand (Figure 2b), while the other one is composed of new-12 and new-22 in the reverse strand (Figure 2c). Multiple sequence alignments of the precursors of these novel miRNAs showed that they contained similar sequence at the ‘seed region’ [Additional file 4: Figure S2], which indicated that they may play similar roles in target regulation and belong to the same family.

### Comparison of miRNA families of *E. granulosus*with other flatworms

**Figure 3:**
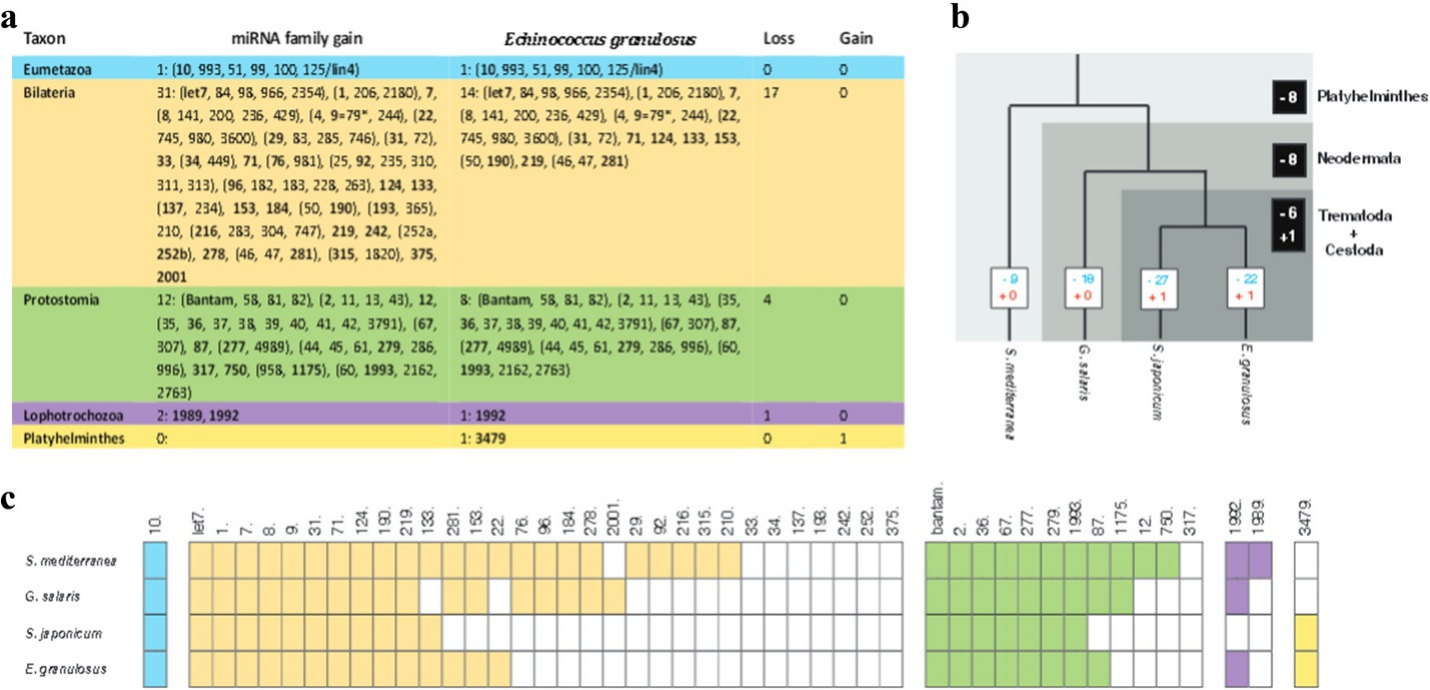
**Loss and gain of miRNA families in** ***E. granulosus*** **and other flatworms** ***.*** **(a)** The table for conserved miRNAs found in *E. granulosus* and inferred loss/gain number of conserved miRNA families. **(b)** The acquisition (red) and loss (blue) of miRNA families for 4 flatworms. Numbers in white squares show evolutionary acquisition and loss of miRNA families in each flatworms; Numbers in black squares show acquisition and loss of miRNA families in flatworms, Neodermataand Trematoda + Cestoda. **(c)** The distribution of the conserved eumetazoan (blue), bilaterian (orange), protostomian (green), lophotrochozoan (purple), and Platyhelminthes (yellow) miRNA families in flatworms.

It is now well established that many miRNAs come in families with the same seed sequences (typically defined as position 2–7 or 2–8 from the 5′ end of the mature miRNA) [30, 31]. From our 94 novel and 23 known miRNAs, 25 different miRNA families representing 29 miRNAs were classified. Of these, 24 families had been expected in the flatworms [8, 9], including one which is characteristic for Eumetazoa, 14 from Bilateria, 8 from Protostomia and one from Lophotrochozoa (Figure 3a). This result was much more than the 16 miRNA families finding in a study by Fromm *et al.* [9]. Interestingly, the remaining one miRNA family (mir-3479) is only present in *E. granulosus, S. mansoni* and *S. japonicum*.

We then compared the presence or absence of the conserved miRNA families of *E. granulosus* (Cestoda) with three other flatworms, *S. mediterranea* (Turbellaria) [18], *Gyrodactylus salaris* (Monogenea) [9] and *S. japonicum* (Trematoda) [22]. Similar to the previous study [9], 8 conserved bilaterian, protostomian, and lophotrochozoan miRNA families were not detected in the four flatworms (Figure 3b and c); these were mir-33, mir-34, mir-137, mir-193, mir-242, mir-252, mir-137 and mir-375. Furthermore, 8 miRNAs families, mir-29, mir-92, mir-216, mir-210, mir-315, mir-12, mir-750 and mir-1989 were only detected in the free living flatworm *S. mediterranea*, but not in *G. salaris*, *E. granulosus* and *S. japonicum*. Finally, there are 6 miRNA families (mir-76, mir-96, mir-184, mir-278, mir-2001 and mir-1175) that are present in *S. mediterranea* and *G. salaris*, but are absent in *S. japonicum* and *E. granulosus*.

### Expression profiles of miRNAs in the three life stages of *E. granulosus*

Combining all detected known and novel miRNAs, 117 pre-miRNAs coding 114 mature miRNAs and 62 miRNA stars were identified in all the *E. granulosus* libraries [Additional file 1: Table S7]. We used the ‘transcripts per million’ (TPM) approach to normalize the abundance value of each miRNA [Additional file 1: Table S8] [32, 33]. Through further evaluating the relative abundance of the mature miRNAs, we found miR-71 was the most abundant miRNA with over 300,000 reads in each library (Table 1), which was similar to the miRNA patterns in *S. japonicum* [34] and *C. elegans* [35]. Whereas most of the conserved miRNAs were expressed constitutively at all development stages, more non-conserved miRNAs were stage-specifically expressed in *E. granulosus*.

**Table 1:** **The relative abundance of the top ten mature miRNAs in three life-stages of** ***E. granulosus***

Name	Adult*	Cyst*	PSC*	Total
**egr-miR-71**	325369.55	546105.37	302855.40	1174330.33
**egr-miR-1**	279745.11	71718.95	142856.71	494320.77
**egr-miR-7**	16646.88	215441.80	10754.68	242843.36
**egr-let-7**	13664.44	31789.98	95330.97	140785.39
**egr-miR-10**	77170.13	18409.03	30980.17	126559.32
**egr-miR-4988**	10474.34	12561.26	95883.37	118918.97
**egr-bantam**	31467.55	23201.17	47866.69	102535.41
**egr-miR-9**	8397.63	9500.11	78860.01	96757.75
**egr-miR-61**	25468.42	10554.49	39333.51	75356.42
**egr-miR-87**	17502.57	19390.63	34640.86	71534.06

*The relative expression levels of each miRNA were calculated by counting the numbers of respective miRNA reads normalized to the total number of reads of annotated miRNAs from each library.

**Figure 4:**
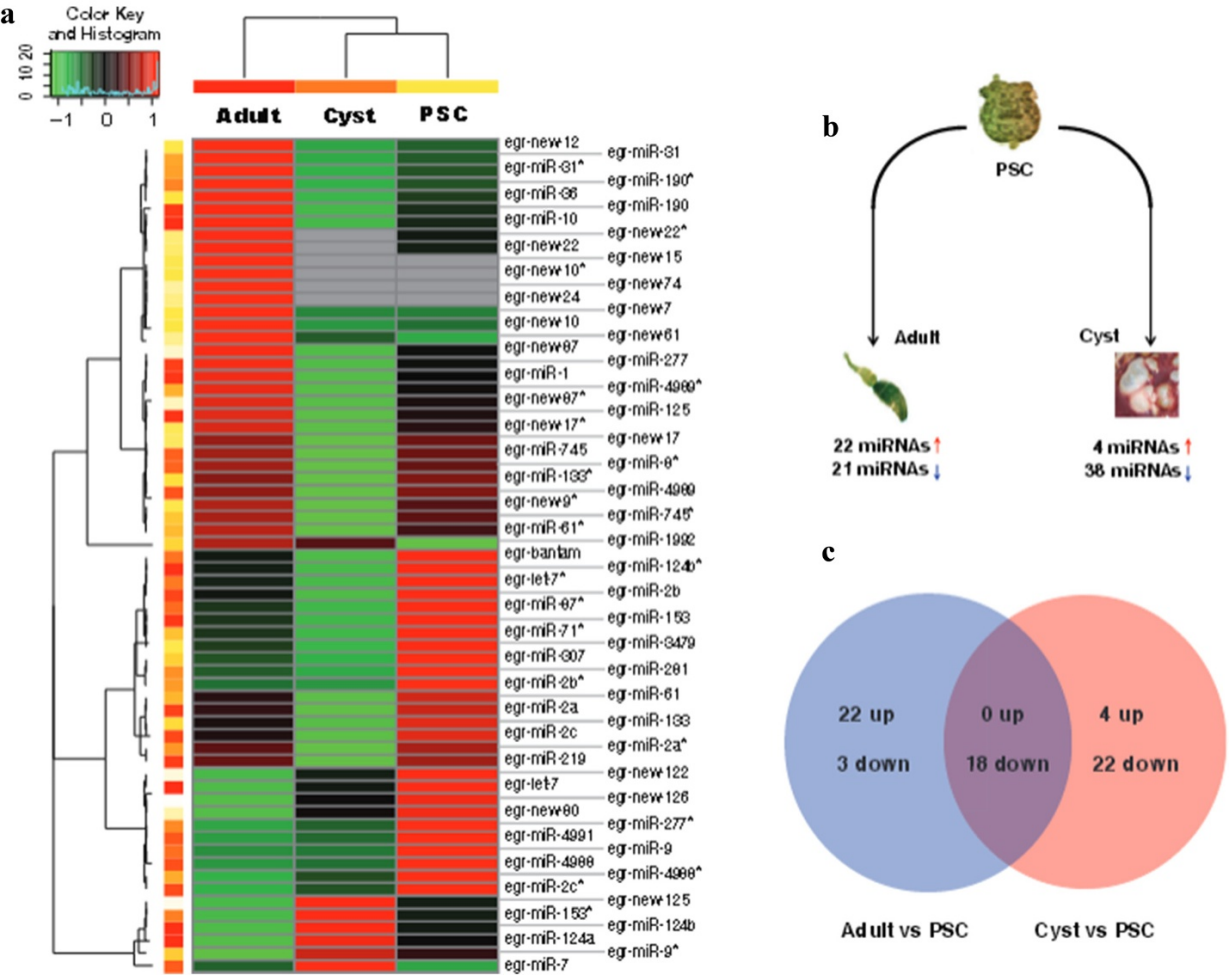
**Different expression profiles of miRNAs in** ***E. granulosus*** **. (a)** Heat maps of sequencing data from 65 differentially expressed miRNAs in the three libraries. The correct *P*-value < 0.001 and fold-change > 2.0 were used as the threshold criteria to define significant differences in miRNA expression. MiRNA expression is displayed using a color key where green corresponds to low and red to high numbers of miRNA normalized reads. **(b)** Differentially expressed miRNAs through comparing the adult or cyst with the PSC. Red arrow indicates the up-regulated miRNAs, and blue arrow represents the down-regulated miRNAs. **(c)** The different expression patterns of miRNAs through comparing the adult or cyst with the PSC. The comparison between the adult and cyst was made and removed similar trends of miRNAs compared to the PSC.

The expression analysis showed that 65 miRNAs, comprising 42 mature miRNAs and 23 miRNA stars, exhibited statistically significant changes (a threshold of correct *P*-value < 0.001 and fold-change > 2.0) in at least one of the three life-stages during *E. granulosus* development (Figure 4a) [Additional file 1: Table S9]. Further evaluation of the expression patterns indicated that 9 miRNAs were mainly expressed in the adult worms and six of them (new-7, new-12, new-15, new-22, new-24 and new-61) are derived from the two new miRNA clusters [Additional file 5: Figure S3]. We used stem-loop real-time quantification RT-PCR to examine the expression of 35 randomly selected mature miRNAs and miRNA stars and 31 miRNAs were shown to be in accordance with the expression profiles detected by the Illumina sequencing [Additional file 1: Table S10].

Since the larval PSC is an important transition life cycle stage, capable of developing either into an adult worm in the dog gastrointestinal tract or a secondary hydatid cyst in the intermediate host, we compared the expression levels of miRNAs in adult worms or cyst membrane with those in the PSC. A total of 43 miRNAs (22 up-regulated and 21 down-regulated) in adult tissue and 42 miRNAs (4 up-regulated and 38 down-regulated) in the cyst membrane were identified (Figure 4b). Further removing similar trends of miRNA expression between adults vs PSC and cyst vs PSC, we found that 25 miRNAs (22 up-regulated and 3 down-regulated) and 24 miRNAs (4 up-regulated and 20 down-regulated) were specifically changed in the adult and cyst stage, respectively (Figure 4c). Amongst these, 8 miRNAs appeared to show reverse trends in expression in the adult and cyst stages compared with PSC; miR-125, miR-277, miR-4989*, new-17*, new-87 and new-87* were up-regulated in adult worms but down-regulated in cyst membrane. In contrast, miR-124a and miR-124b were down-regulated in the former stage but up-regulated in the latter [Additional file 1: Table S11]. These results imply that miRNA expression variation in the different life stages may be associated with the direction of development of PSC into either an adult worm or a secondary hydatid cyst.

### Target gene prediction and functional analysis

To assign biological functions to the 65 differentially expressed miRNAs, we predicted putative target genes using the Miranda program [36]. Based on previously published EST sequences [16], we found 3,622 genes possibly targeted by 114 mature miRNAs and 62 miRNA stars [Additional file 1: Table S12]. By enrichment analyses on the predicted targets, we determined that 182 genes could be categorized into 24 significant GO terms (adjust P < 0.05) [Additional file 1: Table S13]. The major target genes in the biological process categories were involved in the regulation of cell differentiation (GO:0045595), determination of adult lifespan (GO:0008340) and response to nutrient (GO:0007584). These results support our premise that the differentially expressed miRNAs may have important functions in the three different life cycle stages of *E. granulosus*.

**Figure 5:**
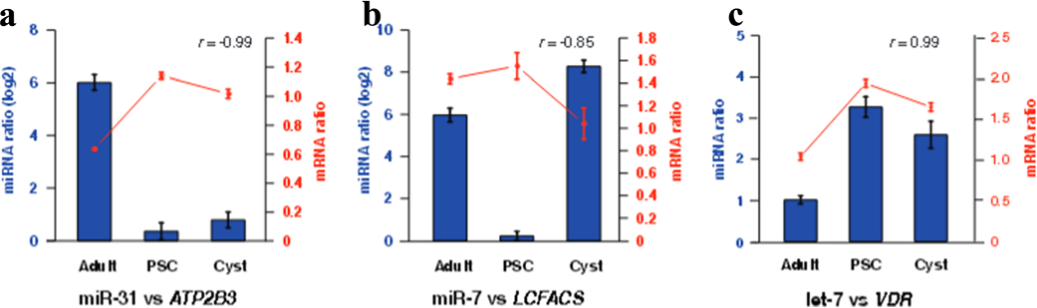
**Comparison of mRNA expression levels of predicted miRNA targets in different stages of** ***E. granulosus*** **. (a)** miR-31 *vs ATP2B*3, **(b)** miR-7 *vs LCFACS* showed nagetive correlation between expression of miRNA and their putative target genes, **(c)** let-7 *vs VDR* showed an positive correlation. The left and right Y-axes indicate the expression levels of the miRNAs and their targets, respectively. Blue bars show the expression levels of the miRNAs (log2 scale) in adult worms (Adult), protoscoleces (PSC) and cyst germinal membranes (Cyst) of *E. granulosus*. Red dots lines represent the expression levels of related target genes.

By using real-time quantitative PCR, we observed that some differentially expressed miRNAs had obvious negative correlations with their target gene expression. For example, a high expressed miR-31 and a down-regulation of its target gene *ATP2B3 (EG_03132)* were simultaneously detected in the adult stage (Figure 5a). Moreover, in the adult and cyst stages of *E. granulosus*, obvious negative correlations were also observed between the expression of miR-7 and its putative target gene long-chain acyl-CoA synthetase (*LCFACS*, EG_02438) (Figure 5b). Finally, we observed a clear positive correlation between the expression of let-7 and its putative target gene vitamin D receptor (*VDR*, EG_04794) (Figure 5c).

## Discussion

In the current study, we performed deep sequencing of small RNAs in three distinct developmental stages of *E. granulosus*. By Bowtie mapping, there are 11,680,028, 12,966,593 and 9,375,095 18–30 nt reads were located the *E. granulosus* genome, respectively. The remaining 40% unmapped reads could be ascribed to the following reasons: (1) some genetic polymorphisms may occur in the *E. granulosus* genome which interferes with Bowtie mapping; (2) unsatisfactory available genome data; some sequences are probably absent in the current draft genome of *E. granulosus* [16]; (3) miRNAs sequencing errors. NGS may randomly produce some base calling errors and influence the accuracy of short reads; (4) post-transcriptional editing of miRNAs may also have contributed to the mismatches observed.

It has been previously reported that the gain of miRNA genes in Metazoan species is associated with an increase in morphological complexity [6]. However, a high rate of the loss of conserved miRNA loci has been found in many subgroups of the Platyhelminthes [9]. Basing on evolutionary conservation analysis, we also found a significant loss of conserved miRNAs in the Cestoda. A total of 22 conserved miRNA families were confirmed as not detectable in *E. granulosus*, suggesting that the loss of conserved miRNA loci in flatworms is not a random event in metazoan phylogenetics. It might relate to loss of targets in the corresponding species [37] or reduced morphological complexity [38]. A previous study revealed that miRNA invention is closely related to the evolution of tissue identities in bilaterian species [7]. Through comparing these lost miRNA families in *E. granulosus*, we found 8 are not detected in the parasitic Platyhelminthes compared with *S. mediterranea* and 6 are absent from *S. japonicum* and *E. granulosus*. On further analysis of their evolutionary origin, we observed that most of the lost miRNAs may be derived from some specific tissues, which have been reduced or lost in tapeworms. For example, there are 8 conserved miRNA families expressed in the free-living *S. mediterranea* that are absent in the Neodermata. Among these, miR-12, miR-216 and miR-283 have been found mainly present in locomotor ciliated cells, while miR-29 and miR-92 are expressed in the gut [7]. These organs have gradually disappeared in *E. granulosus* and some of the other parasitic Platyhelminthes groups because of a significant decrease in locomotor and digestive activities associated with the parasitic mode of life. Furthermore, in the Neodermata, there is a significant degeneration of peripheral sensory nervous system elements, such as, the loss of eyes in tapeworms. In bilaterians, miR-183 and miR-263 have been reported to have a conserved affiliation with sensory organ differentiation [7, 39] and miR-184 and miR-278 play essential roles in the development of eyes [40, 41]. These sensory tissue-specific miRNAs are not detected in *S. japonicum* (Trematoda) and *E. granulosus* (Cestoda). Thus, it seems that the loss of miRNA loci in *E. granulosus* may be related to the reduction or loss of some specific tissues in the Cestoda.

Additionally, the biological evolution of one species to another is usually accompanied by the acquisition of new miRNA families in the newly evolved species [42]. Although previous studies did not report there was any miRNA family acquired in the Platyhelminthes [8, 9], we observed that mir-3479 was present in *E. granulosus*, *S. mansoni* and *S. japonicum*. This result may be due to the lack of information in other taxa or due to evolutionary innovations to adapt to the new living environment. For tapeworms, in order to escape from host immune attack, *E. granulosus* produces many components to moderate the host immune response, such as EgAgB (antigen B), which interferes with human dendritic cell differentiation and maturation [43]. Further, one recent study has shown that three schistosome-specific miRNAs, Bantam, miR-10 and miR-3479, were detected in the plasma of *S. japonicum-*infected rabbits [44]. These results imply that the gain of miR-3479 in *E. granulosus* or *S. japonicum* may have resulted as an evolved adaptation in the parasitic Platyhelminthes to moderate the host immune response. It is important to note that there are no reports to date on the plasma levels of *E. granulosus* miRNAs in patients with CE. However, some *Dicrocoelium*-specific miRNAs have recently been shown to be present in exosome-like vesicles [45] and similar secretory vesicles have been described previously in the early stages of *E. granulosus* development [46].

Notably, two novel close miRNA clusters (new-15/ new-24/new-61/new-7 and new-12/new-22) were identified in *E. granulosus* and *E. multilocularis*. Beside the same genomic location, they display similar patterns of miRNA expression, suggesting that the expression of these novel miRNAs may be co-regulated by the same promoter elements. Furthermore, two clusters of miRNAs share the ‘GGCGCUU’ motif with the known sme-miR-2160. Recently, it has been reported that sme-miR-2160 is enriched in planarian stem cells and is down-regulated in individuals in which stem cells had been abrogated by irradiation [18]. These observations imply that these two clusters of miRNAs may be associated in *E. granulosus* with cell proliferation and differentiation. Moreover, since new-7/12/15/22/24/61 of *E. granulosus* contains six copies of the same seed sequences in the 5′ hairpin arms, these multiple copies may likely be functional, resulting in the dose-dependent regulation of their downstream targets [47, 48]. Similar multiple copies of mir-2160 have been identified in *S. mediterranea* [18].

Development in *E. granulosus* involves a number of different life stages including the eggs, activated oncospheres, hydatid cyst, PSC and adult worms. It is a complicated and dynamic process and may be associated with miRNA regulation. By comparing the differential expression pattern of miRNAs, our results showed that let-7 exhibited 3.2-fold and 2.6-fold increased expression in PSC and in the cyst, respectively, compared with the adult. let-7 has been shown to regulate developmental timing in *C.elegans* through the direct target genes *lin-41* and *hbl-1* [49]. Thus, the observation of substantially increased expression of let-7 in the PSC and cyst might be associated with the capability of *E. granulosus* for bi-directional development, differentiating into an adult worm in the definitive host dog gastrointestinal tract or into a secondary hydatid cyst in the intermediate (or human) host [50].

Indeed, bi-directional development is a unique biological characteristic of *E. granulosus* and our previous study showed that some protein-coding genes, with high homology to the bile acid receptors and transporters, were located in the *E. granulosus* genome, and that nuclear hormone receptors, including the farnesoid X receptor (FXR) and VDR, may play a key role in stimulating the PSC to develop into adult worms [16]. Recently, the hormone receptors have been reported to directly activate transcription of the evolutionarily conserved let-7 and control the development of larvae to adults in *C. elegans* [51]. Here, we observed a positive correlation between the expression of let-7 and *VDR*, suggesting that VDR may induce crucial miRNA transcriptional events and promote adult development in *E. granulosus*. Furthermore, through miRNA target prediction analysis, we found let-7 could complementary bind to the *VDR* 3′ UTR sequences, suggesting that let-7 may mediate a negative feedback loop controlling the bile acid signal pathway in the development of PSC. Recently, a feedback circuit between let-7-family miRNAs and DAF-12, a homolog of vertebrate FXR and VDR, has been found in *C. elegans* [52] *.* Additional experimental validations are required to explore this relationship in *E. granulosus* in future studies.

## Conclusions

In summary, this study describes the first large scale identification and dynamic characterisation of miRNAs in three developmental stages of *E. granulosus*. A total of 114 mature miRNAs and 62 miRNA stars were identified. Evolutionary conservation analysis suggested that the losses of miRNA families in *E. granulosus* may be associated with morphological reductions. Additionally, GO analysis revealed that the differentially expressed miRNAs and their targets may be involved in diverse development processes, including bi-directional differentiation and nervous system development. Understanding the regulatory processes involving miRNAs in *E. granulosus* may be helpful to explore the mechanism of interaction between this parasitic worm and its definitive and intermediate hosts, and provide new information to develop new interventions and therapeutics for the control of CE.

## Methods

### Ethics statement

Ethical approval for the use of dogs and sheep was given by the Animal Ethics Committee of Xinjiang Academy of Animal Science(XJAASaec2011031506). The protocols for using dogs and sheep were covered under the < The Guidelines for the Care and Use of Experimental Animals > (China) National Science and Development Act 2006–398.

### Preparation of *E. granulosus*samples

The preparation of *E. granulosus* samples for analysis was undertaken as previously described [53, 54]. PSC were aspirated aseptically from several sheep liver hydatid cysts collected from Xinjiang, China. The PSCs were sedimented at 1000 × *g* for 15 minutes and digested by pepsin [16]. After PSC were removed, cyst membranes were rinsed 10 times with PBS and checked microscopy to confirm no PSC attached on the membranes, and then cut into small pieces with a pair of scissors. The cut membranes were soaked in PBS in a flask and stirred for 10 min at 4°C. The flask was placed on bench at room temperature for 3 min to precipitate laminated layer and other cyst tissues. The membrane cells in supernatant were pelleted by centrifuging at 1400 g for 10 min at 4°C. The cells were resuspended with PBS and left on the bench for 3 min to precipitate remained PSC, the supernatant was centrifuged at 1400 × *g* for 10 min at 4°C. Adult worms were collected from experimentally infected dogs using procedures describe earlier [55]. All parasite materials were subjected to a final wash in PBS and then they were suspended in 10 volumes of RNAlater (Ambion, Austin, TX, USA), and stored at -80°C.

### Total RNA extraction

Samples of the 3 different life cycle stages of *E. granulosus* (adult, cyst and PSC) were homogenized in lysis buffer and total RNA was extracted using mirVana™ miRNA isolation kits (Ambion, Austin, TX, USA), according to the manufacturer’s instructions. RNA concentration and purity were monitored using a NanoDrop ND-1000 UV spectrophotometer (Nanodrop Technologies, Wilmington, DE). The RNA integrity was evaluated by an Agilent 2100 Bioanalyzer (Agilent Technologies, Palo Alto, CA) [Additional file 6: Figure S4]. Finally, all RNA samples were stored at -80°C.

### Small RNA library construction and deep sequencing

We constructed each small RNA library using Illumina Small RNA v1.5 Sample Preparation Kits (Illumina, San Diego, CA). Briefly, total RNA (5 μg) of each sample was resolved on denaturing polyacrylamide gel electrophoresis (PAGE) gels, and then fractions of 18 to 30 nt in size were collected. The isolated small RNAs were sequentially ligated to 3′adapters, using T4 RNA ligase 2 (New England Biolabs, Ipswich, MA, USA), and 5′adapters using T4 RNA ligase (New England Biolabs, Ipswich, MA, USA). The ligation products were reverse transcribed using SuperScript Reverse Transcriptase II (Life Technologies, Gaithersburg, MD, USA) and amplified with 12 PCR cycles. Then, 6% (w/v) PAGE was used to purify the amplification products. Finally, the libraries were used for clustering and sequencing using an Illumina Genome Analyzer II (Illumina, San Diego, CA).

### Computational analysis to search for novel miRNAs and other small RNAs

After removing low quality sequence reads and trimming adaptor sequences through miRExpress [56], we collected small RNAs ranging from 18–30 nucleotides and count length distributions. Then, duplicated sequences were removed from the initial dataset to produce a nonredundant set of unique sequences, hereafter referred to as sequence tags. To determine whether these small RNA sequences were from *E. granulosus*, we mapped these small RNA sequences by Bowtie [57] to the draft *E. granulosus* genome sequences [16]. Only those mapping perfectly onto the draft genome were further considered as candidate miRNAs. If a sequence mapped to more than 10 loci in the genome, it was not considered for further analysis. Then, we compared all sequence tags against a database of known miRNA precursors sequences (http://www.mirbase.org/, release 21.0) [4], and profiled every annotated miRNA in each library.

Basing on our earlier findings [16] and scrutiny of the Rfam RNA family database (http://rfam.sanger.ac.uk), we discarded all sequences matching with known *E. granulosus* rRNAs, tRNAs, snRNAs and mRNAs. With the annotation of repeat sequences in the *E. granulosus* genome, small RNAs positioned at repeat loci were identified and annotated as repeat-associated small RNAs. Finally, the remaining clean sequenced reads were used to search for both conserved and novel miRNAs.

In order to identify new miRNAs, we used miRDeep 2.0 [27] to identify miRNAs without prior information. The software uses a Bayesian algorithm and provides a score based on the expected pattern of miRNA-duplex excision from the stem of the precursor hairpins by Dicer [58]. The secondary structures of putative precursors were identified using the RNAfold program [59] and computed by minimum free energy (MFE). We used the following criteria to filter the candidate miRNAs: (1) The lengths of mature miRNA ranged from 18 to 25 nucleotides; (2) The miRNA precursors should have a characteristic fold-back structure and MFE should be less than -20 Kcal/mol; (3) The miRDeep score should be more than 1; (4) The GC content of the mature miRNA should be 15%-85%; (5) Small RNAs with multiple loci in the *E. granulosus* genome were excluded (more than five times); (6) In order to remove accidental hits, only the precursors with at least 5 sequence reads in three libraries were used. Furthermore, these predicted mature miRNAs were also compared with known miRNAs from other species. If the *E. granulosus* miRNAs shared >80% homology with known miRNAs and had the same seed sequences (2–8 nt), the miRNAs were considered as conserved miRNAs and were thus named after the known miRNA [60]. We defined the mature miRNA and miRNA star basing on the total number of reads in three life stages.

### Detection of differential expression

Using the quantifier module of miRDeep2, we summed up read counts for all known or novel mature miRNAs in the sequencing data. Then, relative expression levels were calculated by counting the numbers of respective miRNA reads normalized to the total number of annotated miRNAs in each library. After ignoring the simultaneously lowly expressed miRNAs in three libraries (less than 50 TPM at all stages), differential expression in the three libraries was evaluated using the statistical R package DEGseq [61]. (http://www.bioconductor.org/packages/2.6/bioc/html/DEGseq.html). Based on this statistical model, statistical significances were calculated using Fisher’s exact test and likelihood ratio test, and then corrected for multiple testing according to Benjamini-Hochberg multiple test method [62]. Potentially interesting miRNA candidates were chosen according to the criteria of a 2-fold expression level change and adjusted *P*-value < 0.001.

### MicroRNA target prediction

In order to seek miRNAs targets, we first predicted the 3′UTRs by combined analysis of the predicted mRNAs and the released EST scafford sequences for *E. granulosus* [16]. Only the sequences located at the 3′untranslated region of predicted genes were regarded as potential 3′UTR sequences. Then, the miRanda program [36] was used to predict the target genes for all mature miRNAs. The energy thresholds were set at ≤ -20 kcal/mol and other thresholds used a default value (score threshold, 120; gap-opening penalty, -9; gap-extend penalty, -4). Then, all ESTs sequences predicted to contain miRNA target sites, were annotated as earlier described [16].

### Gene ontology and pathway analysis

Target genes regulated by the different subgroups of miRNAs were collected and subjected to Gene Ontology (GO) and Kyoto Encyclopedia of Genes and Genomes (KEGG) pathway analysis. Based on NCBI-nr annotation, we used the BLAST2GO program (http://www.BLAST2go.org/) to obtain GO annotation of every target [63]. Then, the hypergeometric test [64] was used to classify the GO category, and the false discovery rate (FDR) was calculated to correct the *P*-value. KEGG pathway analysis was carried out using the KEGG Automatic Annotation Server for ortholog assignment and pathway mapping (http://www.genome.jp/tools/kaas) [65]. The hypergeometric test was used to assess the significant pathway of enrichment.

### Quantitative RT-PCR of miRNAs and their targets

A stem-loop quantitative RT-PCR was performed to validate the miRNA expression levels in each developmental stage. Total RNA was extracted using mirVana^TM^ miRNA isolation kits (Ambion, Austin, TX, USA) according to the manufacturer’s instructions. The total RNA was reverse transcribed in a 20 μl reverse transcription (RT) reaction using 500 ng total RNA, 1 U PrimeScript RT Enzyme Mix I (Takara Bio, Otsu, Japan), 4 μl of 5 × RT buffer, and 1 μl of 2 μM miRNA-specific stem-loop primers [Additional file 1: Table S14a] designed according to Chen *et al.* [28]. 5.8S rRNA was chosen as a reference and was reverse-transcribed with a specific reverse primer [Additional file 1: Table S14a]. The RT reaction mixture was incubated at 37°C for 30 minutes, then at 85°C for 5 seconds. A control was set up at the same time with no RNA input. With the cDNA products as a template, quantitative PCR was carried out using SYBR® Premix Ex Taq^TM^ (Takara Bio, Otsu, Japan) in a StepOne Plus real-time system (Applied Biosystems, Carlsbad, CA, USA). The primer sequences are listed in [Additional file 1: Table S14a and S14b]. A 10 μl reaction mixture including 1 μl of diluted cDNA (1:5), 0.4 μl of each primer (10 mM), 5 μl SYBR Premix Ex Taq II and 3.6 ml H_2_O was placed in 0.2 ml eight-strip PCR tubes (Applied Biosystems, Carlsbad, CA, USA). Cycling conditions were: 95°C for 30s, followed by 40 cycles of 95°C for 5 s and 60°C for 34 s, 72°C for 60s. For each PCR, dissociation curve analysis was carried out to discriminate the specific products from the primer dimers. The CT-values are the average of three technical and three biological replicates and fold changes of miRNAs and their targets in different samples were calculated by the 2-ΔΔCt method.

### Statistical analysis

The miRNA expression levels in each developmental stage were compared by using a two-tailed *t*-test. Correlation analysis was made using the Pearson correlation coefficient. P < 0.05 was considered statistically significant.

## Electronic supplementary material

Additional file 1: **A file of all supplementary tables in the present study.** **Table S1.** Length distribution of the sequencing reads in three libraries of *E. granulosus.* **Table S2.** Raw data filtration and distribution of sequenced small RNAs across different categories. **Table S3.** All identified known mature miRNAs and miRNA stars. **Table S4.** All predicted novel miRNA precursors in *E. granulosus*. **Table S5.** All identified novel mature miRNAs and miRNA stars. **Table S6.** The conservation analysis of the *E. granulosus* miRNA candidates in present *E. multilocularis.* **Table S7.** Summary of all previously known and newly found miRNAs in *E. granulosus.* **Table S8.** Profiles of miRNAs from adult, protoscolex (PSC) and the cyst of *E. granulosus*. **Table S9.** The miRNAs with different expression patterns in the different development stages of *E. granulosus.* **Table S10.** Validation of miRNA expression patterns by stem-loop real-time quantitative PCR. **Table S11.** The different expression patterns of miRNAs in the adult or cyst compared with PSC. **Table S12.** Target genes of all detected miRNAs. **Table S13.** Gene Ontology (GO) abundance analysis of the putative target genes for the differentially expressed miRNAs in *E. granulosus.* **Table S14.** Sequences of stem-loop RT primers, forward primers and reverse primer. (XLS 3 MB)

Additional file 2: **The secondary structure predictions and homology sequence alignments of all predicted miRNAs.** (PDF 218 KB)

Additional file 3: Figure S1: Confirmation of expression of novel miRNAs using stem-loop PT PCR. (PDF 443 KB)

Additional file 4: Figure S2: Sequence alignment of six new *E. granulosus* miRNAs in two newly found cluster. (PDF 262 KB)

Additional file 5: Figure S3: Stem-loop semi-quantitative PCR of six new *E. granulosus* miRNAs with 5.8 s rRNA as an internal control. (PDF 294 KB)

Additional file 6: Figure S4: The RNA integrity (RIN) of the Samples of the 3 different life cycle stages. (PDF 298 KB)

## Abbreviations

miRNAs: microRNAs
*E. granulosus*: *Echinococcus granulosus*
*E. multilocularis*: *Echinococcus multilocularis*
CE: Cystic echinococcosis
PSC: Protoscolex
GO: Gene ontology
*ATP2B3*: Plasma membrane calcium-transporting atpase 3-like
*LCFACS*: Long-chain acyl-CoA synthetase
MFE: Minimum free energy
3′UTR: 3′-untranslational regions
FDR: False discovery rate
TPM: Transcripts per million
KEGG: Kyoto Encyclopedia of Genes and Genomes.
