# Supplementary material for: Genome-wide sequencing of small RNAs reveals a tissue-specific loss of conserved microRNA families in Echinococcus granulosus
Source: BMC Genomics. 2014 Aug 29;15(1):736. doi: 10.1186/1471-2164-15-736 (PMC4156656; doi:10.1186/1471-2164-15-736)
Supplement: Supplementary file 2 — Additional file 2: The secondary structure predictions and homology sequence alignments of all predicted miRNAs. (PDF 218 KB) [file 12864_2014_6407_MOESM2_ESM.pdf]

egr-bantam

hairpin:
CGGCUUUUCUCGCGUUCUGAGAGCGUUAAUUCUUUAGUUAAACUACCGCCUUCUGAGAUCGCGAUUACAGCUGAU
mature:
UGAGAUCGCGAUUACAGCUGAU

ENERGY = -23.5

total reads	mature	loop	star
375292	374999	15	278

Bases 1 to 75

```
          10        20               30
--     UUUC     U   G   --   -----|    UC
  CGGCU    UCGCG UCU AGA  GCG     UUAAU  U
  GUCGA    AGCGC AGA UCU  CGC     AAUUG  U
UA     CAUU     U   G   UC   CAUCA^    AU
    70        60        50        40
```

```
                     *******      * ****
sja-bantam           UGAGAUCGCGAUUAAAGCUGGU
sma-bantam           UGAGAUCGCGAUUAAAGCUGGU
egr-bantam           UGAGAUCGCGAUUACAGCUGAU
sme-bantam-b-3p      UGAGAUCACUGCGAAAGCUGAU
sme-bantam-a         UGAGAUCACUAUGAAAGCUGG-
sme-bantam-c-3p      UGAGAUCAUUAUGAAAGCUUUU
```

---

egr-miR-61

hairpin:
CGUGAGGCCCUUUCUUGUGCAUGGAUAAUGAGGGGUCUUUAAGCCUCGACCAUGACUAGAAAGAGCACUCACAUCC
mature:
UGACUAGAAAGAGCACUCACAUCC

ENERGY = -30.5

total reads	mature	loop	star
273328	272555	1	772

Bases 1 to 76

```
            10        20        30
C---     -| C      U  G     AUAAUGA     CU
    GUGAG GC CUUUCU GU CAUGG       GGGGU  U
    CACUC CG GAAAGA CA GUACC       CUCCG  U
CCUA     A^ A      U  -     AG-----     AA
     70        60         50             40
```

```
                     ********  *   **
egr-miR-61           UGACUAGAAAGAGCACUCACAUCC
sja-miR-61           UGACUAGAAAGUGCACUCACUU--
sma-miR-61           UGACUAGAAAGUGCACUCACUU--
cte-miR-2720-3p      UGACUAGAGAGUUUACUCAUCC--
sme-miR-61a-3p       UGACUAGAAAGUUCACUUACUGU-
sme-miR-61b-3p       UGACUAGAAUGUUCACCUUCUUU-
```

---

egr-miR-31

hairpin:
UGGCAAGAUACUGGCGAAGCUGAGGGGUCAAGUAAUCGUCAGCUUCGUCUGGUCUUGCUGC
mature:
UGGCAAGAUACUGGCGAAGCUGA

ENERGY = -32.4

total reads	mature	loop	star
126020	124603	48	1369

Bases 1 to 61

```
         10        20        30
U-|       ACU          G  GUCA
  GGCAAGAU   GGCGAAGCUGA GG    \
  UCGUUCUG   CUGCUUCGACU CU    A
CG^       GU-          G  AAUG
60        50          40
```

```
                     ********   ***  * **
cte-miR-31          AGGCAAGAUGUUGGCAUAGCU--
lgi-miR-31          AGGCAAGAUGUUGGCAUAGCU--
sme-miR-31a-5p      AGGCAAGAUGUUGGCAUAACUGA
sme-miR-31b-5p      AGGCAAGAUGCUGGCAUAGCUGA
sma-miR-31-5p       UGGCAAGAUUAUGGCGAAGCUGA
sja-miR-31-5p       UGGCAAGAUUACGGCGAAGCUGA
egr-miR-31          UGGCAAGAUACUGGCGAAGCUGA
```

---

egr-miR-2162

hairpin:
AGUGGAUUUGUUGCAUAUUAUACAUCGUGGGGUUUGUAUUAUGCAACUUUUCACUCC
mature:
UAUUAUGCAACUUUUCACUCC

ENERGY = -20

total reads	mature	loop	star
36158	31005	0	5153

Bases 1 to 57

```
          10        20
--|     UUU        U    C   G
  AGUGGA   GUUGCAUA UAUA AUC U
  UCACUU   CAACGUAU AUGU UGG G
CC^     UU-        U    U   G
      50         40        30
```

```
                     ********     *****
cte-miR-1993         UAUUAUGCUGAUAUUCACGAGA
lgi-miR-1993         UAUUAUGCUGAUAUUCACGAGA
sme-miR-2162-3p      UAUUAUGCAAAUAUUCACAAU-
egr-miR-2162         UAUUAUGCAACUUUUCACUCC-
sja-miR-2162-3p      UAUUAUGCAACGUUUCACUCU-
```

---

egr-miR-3479

hairpin:
CGGUGAAAGUUUAUGCAUUUACAUCGGUUGAUGUAUUGCACGUUCUUUCGCCAUC
mature:
UAUUGCACGUUCUUUCGCCAUC

ENERGY = -23.3

total reads	mature	loop	star
19325	16950	11	2364

Bases 1 to 55

```
          10        20
C--|       UUUA    UU      G
   GGUGAAAG    UGCA  UACAUC G
   CCGCUUUC    ACGU  AUGUAG U
CUA^       UUGC    U-      U
    50        40         30
```

```
                     ********   * ****** *
sja-miR-3479-3p      UAUUGCACUUACCUUCGCCUUG
sma-miR-3479-3p      UAUUGCACUAACCUUCGCCUUG
egr-miR-3479         UAUUGCACGUUCUUUCGCCAUC
```

---

egr-miR-281

hairpin:
AAGGAGGGCCCUUUAUGACCCUUGAGAAAUAAAUCAAUGUCAUGGAGUUGCUCUCUAUA
mature:
UGUCAUGGAGUUGCUCUCUAUA

ENERGY = -22.5

total reads	mature	loop	star
11111	11061	0	50

Bases 1 to 59

```
         10         20
AA-       C-|        CC     GAA
   GGAGGGC  CUUUAUGAC  UUGA    A
   UCUCUCG  GAGGUACUG  AACU    U
AUA       UU^        U-     AAA
        50        40         30
```

```
                    *********  ******
cte-miR-281         UGUCAUGGAGUUGCUCUCUUUA
lgi-miR-281-3p      UGUCAUGGAGUUGCUCUCUUUA
egr-miR-281         UGUCAUGGAGUUGCUCUCUAUA
sme-miR-281-3p      UGUCAUGGAUAUGCUCUUC---
```

---

egr-miR-36

hairpin:
ACGGAUUGUCCGUCCGGUAACCGCGAUAAGGUGAAGGCCUUUUUAACUGCUGGUCACCGGGUAGACAUUCCUUGC
mature:
UCACCGGGUAGACAUUCCUUGC

ENERGY = -31.6

total reads	mature	loop	star
2901	2896	2	3

Bases 1 to 75

```
          10         20              30
AC--    U    CG       A    -    AU----|     G
    GGA UGUC  UCCGGU ACC GCG       AAGGU A
    CCU ACAG  GGGCCA UGG CGU       UUCCG A
CGUU    U    AU       C    U    CAAUUU^     G
    70        60        50        40
```

```
                     ********  *****  *
egr-miR-36          UCACCGGGUAGACAUUCCUUGC-
sme-miR-36c-3p      UCACCGGGUAGACAUUCAUU---
sja-miR-36-3p       CCACCGGGUAGACAUUCAUUCGC
sma-miR-36-3p       CCACCGGGUAGACAUUCAUUCGC
sme-miR-36a-3p      UCACCGGGUAGACAUUCAUUA--
sme-miR-36b-3p      UCACCGGGUAGACAUUAAUCAUG
cte-miR-36          UCACCGGGUUAACAUUCAUCCG-
```

---

egr-miR-133

hairpin:
GGCUGAUUUGUGGGGCUCAGAAAAGCUGUAAAUGAAAACUUUUGGUCCCCAUUAACCAGCCGC
mature:
UUGGUCCCCAUUAACCAGCCGC

ENERGY = -22.9

total reads	mature	loop	star
1749	1044	19	686

Bases 1 to 63

```
          10        20        30
--|    A  U      CU     AAGCUGUA
  GGCUG UU GUGGGG  CAGAA        \
  CCGAC AA UACCCC  GUUUU        A
CG^    C  U      UG     CAAAAGUA
  60        50        40
```

```
                     ********  * *******
cte-miR-133          UUGGUCCCCUUCAACCAGCUGU
lgi-miR-133-3p       UUGGUCCCCUUCAACCAGCUGU
sme-miR-133a-3p      UUGGUCCCCGUCAACCAGCUGU
sja-miR-133          UUGGUCCCUAUCAACCAGCUGU
sme-miR-133b-3p      UUGGUCCCCAUCAACCAGCA--
egr-miR-133          UUGGUCCCCAUUAACCAGCCGC
```

---

egr-miR-1992

hairpin:
UUUCAUUGGUCAAUUGCUAAUAUUUCUUGCGUAAAUUUACAAAUAUCAGCAGUUGUACCAUUGAAAUG
mature:
UCAGCAGUUGUACCAUUGAAAUG

ENERGY = -21.3

total reads mature loop star
1519 1218 1 300

Bases 1 to 68

```
          10         20        30
--     U    -|       A      CUUGC   A
  UUUCA UGGU CAAUUGCU AUAUUU     GUA A
  AAAGU ACCA GUUGACGA UAUAAA     CAU U
GU     U    U^       C      -----   U
       60        50              40
```

```
                     *********** *** ***
cte-miR-1992         UCAGCAGUUGUACCACUGAUGUG-
cla-miR-1992         UCAGCAGUUGUACCACUGAUGUGU
lgi-miR-1992         UCAGCAGUUGUACCACUGAUUUG-
egr-miR-1992         UCAGCAGUUGUACCAUUGAAAUG-
sme-miR-1992-3p      UCAGCAGUUGUUCCAUUGAC----
```

---

egr-miR-307

hairpin:
AUCCCGUGGGUUGAGCGAUGCCUUUGUGUUCUGCACCUCUAUGCGCAUCACAACCUACUUGAUUGAGG
mature:
UCACAACCUACUUGAUUGAGG

ENERGY = -20.4

total reads mature loop star
40982 40877 10 95

Bases 1 to 69

```
             10        20            30
-----   CC        AGC      CUUU----|  UU
     AUC  GUGGGUUG   GAUGC         GUG  \
     UAG  CAUCCAAC   CUACG         CAC  C
GGAGU   UU        A--      CGUAUCUC^  GU
       60        50          40
```

```
                   ********* * ***  ***
egr-miR-307        UCACAACCUACUUGAUUGAGG--
sja-miR-307        UCACAACCUACUUGAUUGAG---
cte-miR-67         UCACAACCUGCAUGAAUGAGGU-
lgi-miR-67         UCACAACCUGCAUGAAUGAGGAC
sme-miR-67-3p      UCACAACCUCCAUGAACGAGGGU
```

---

egr-new-7

hairpin:
UGGCGCUUUCUAACUUUACUGAUAGUGAUUUAUCAGUAAAGUCAUAAAUGCACCUCA
mature:
UGGCGCUUUCUAACUUUACUGA

ENERGY = -19.7

total reads	mature	loop	star
37789	37786	0	3

Bases 1 to 57

```
           10        20
U--  C  -|  CUA            U
   GG GC UUU   ACUUUACUGAUAG G
   CC CG AAA   UGAAAUGACUAUU A
ACU  A  U^  UAC            U
      50        40        30
```

NA

---

egr-new-10

hairpin:
UGAGUAUUGUACCAUGCGGUGAGUGACCUUGUCUCCUUCCACCUACUCAUUGCAGGGUAGAAUACUCC
mature:
UGAGUAUUGUACCAUGCGGUGA

ENERGY = -28.9

total reads	mature	loop	star
17014	16815	4	195

Bases 1 to 68

```
        10        20        30
U|      G    A          ACCUUGUC
 GAGUAUU UACC UGCGGUGAGUG        U
 CUCAUAA AUGG ACGUUACUCAU        C
C^      G    G          CCACCUUC
       60        50        40
```

NA

---

egr-new-12

hairpin:
UGGCGCUUGAUUUCAACACUGUUAGAAGCAACAGUGUUGAUACCAGGCAUUCACC
mature:
UGGCGCUUGAUUUCAACACUGU

ENERGY = -22.3

total reads	mature	loop	star
4850	4845	0	5

Bases 1 to 55

```
           10        20
UGGC---|    AUU           AG
       GCUUG   UCAACACUGUU  A
       CGGAC   AGUUGUGACAA  A
CCACUUA^    CAU           CG
    50        40        30
```

NA

---

egr-new-15

hairpin:
UGGAGCUUGGUGACAAGACUGACAGACAAGUGGCAGUGUUGCUAUCAAUGCUCCGCA
mature:
UGGAGCUUGGUGACAAGACUGAC

ENERGY = -23.3

total reads	mature	loop	star
2445	2444	0	1

Bases 1 to 57

```
           10        20
U--     -|     A   G    A  GA
   GGAGC UUGGUG CAA ACUG CA  C
   CCUCG AACUAU GUU UGAC GU  A
ACG     U^     C   G    G  GA
      50        40        30
```

NA

---

egr-new-17

hairpin:
GGGCUCGUUUACUCGAAUUGGUCUGCGUGGGGCACUAAUUCGAGUCAACAGGGUCGUU
mature:
GGGCUCGUUUACUCGAAUUGGU

ENERGY = -25.1

total reads	mature	loop	star
1814	1045	0	769

Bases 1 to 58

```
           10        20
G--     -|  U            CU  GU
   GGCUC GUU ACUCGAAUUGGU  GC  \
   CUGGG CAA UGAGCUUAAUCA  CG  G
UUG     A^  C            --  GG
       50        40          30
```

NA

---

egr-new-22

hairpin:
UGGCGCUUGAUUUCAACGCUGAUAGAAAACACCGUACAUCAGUGUUGAUGUCAGGUUGCUUCU
mature:
AGUGUUGAUGUCAGGUUGCUUCU

ENERGY = -24.9

total reads	mature	loop	star
1180	1079	5	96

Bases 1 to 63

```
           10        20        30
U--   -|      U           AGAAAA
   GGC GCUUGAU UCAACGCUGAU      C
   UCG UGGACUG AGUUGUGACUA      A
UCU   U^      U           CAUGCC
  60        50        40
```

NA

---

egr-new-24

hairpin:
UGGCGCUUGACCAUGCUACUGAUAGACGCAAAUCAGUUGUAUCAUCAAUGCGCCGCA
mature:
UGGCGCUUGACCAUGCUACUGA

ENERGY = -23.7

total reads	mature	loop	star
534	533	0	1

Bases 1 to 57

```
           10        20
U--     -|   CC    U      AGA
   GGCGC UUGA  AUGC ACUGAU   C
   CCGCG AACU  UAUG UGACUA   G
ACG     U^   AC    U      AAC
      50        40        30
```

NA

---

egr-new-25

hairpin:
UAUAUCAAAACGUCAGGUUUUGGUGGAAAAGAAAACAUGGCGUUUUGGUAAGA
mature:
AACAUGGCGUUUUGGUAAGA

ENERGY = -20.5

total reads	mature	loop	star
518	335	0	183

Bases 1 to 53

```
         10        20
UA-|            G     GGUG
   UAUCAAAACGUCA GUUUU    G
   AUGGUUUUGCGGU CAAAA    A
AGA^            A     GAAA
  50        40        30
```

NA

---

egr-new-26

hairpin:
GGGUCAUUCUGAUAGUUCAGAUAAACGGGUCAGAACUAUCGGAAUGGCAGU
mature:
CAGAACUAUCGGAAUGGCAGU

ENERGY = -26

total reads	mature	loop	star
245	239	0	6

Bases 1 to 51

```
         10        20
GG-|               A   AA
   GUCAUUCUGAUAGUUC GAU  A
   CGGUAAGGCUAUCAAG CUG  C
UGA^               A   GG
50        40        30
```

NA

---

egr-new-28

hairpin:
CCUCCAUUUCCGGUUCGAAUGGGCGCAUAACUACCGCUCGAACUGGGGUUGAAGC
mature:
GCUCGAACUGGGGUUGAAGC

ENERGY = -20

total reads	mature	loop	star
176	170	0	6

Bases 1 to 55

```
        10        20
C| C  U           A   GCGCA
 CU CA UUCCGGUUCGA UGG     \
 GA GU GGGGUCAAGCU GCC     U
C^ A  U           C   AUCAA
    50        40        30
```

NA

---

egr-new-29

hairpin:
UUGCACAGCUUAGAGCUCUGUCUGCUUUUCGCCUGCGGAGUGUCGGUGCUGUGCGAAAGUGAU
mature:
UUGCACAGCUUAGAGCUCUGUC

ENERGY = -27.7

total reads	mature	loop	star
130	129	0	1

Bases 1 to 63

```
               10         20
-------         UUA  -|      CU  UU
       UUGCACAGC   GA GCUCUGU  GC  \
       AGCGUGUCG   CU UGAGGCG  CG  U
UAGUGAA         UGG  G^      UC  CU
  60        50        40        30
```

NA

---

egr-new-33

hairpin:
GGCGACAGACGAUAAUAGACAGGUCGACAAGUAAUGACUUCAGCUUGUCUAUUAGUCAUCUGUCUCAUU
mature:
GGCGACAGACGAUAAUAGAC

ENERGY = -26.9

total reads	mature	loop	star
89	85	0	4

Bases 1 to 69

```
           10         20        30
---| C      C   -           C  CA   A
   GG GACAGA GAU AAUAGACAGGU GA  AGU A
   CU CUGUCU CUG UUAUCUGUUCG CU  UCA U
UUA^ -      A   A           A  --   G
         60        50        40
```

NA

---

egr-new-37

hairpin:
UGAACUCUGUCAUUAAAUUUGCUAGUCGUAGCAAAUGUGUGGACGAGUUCGCGA
mature:
UGAACUCUGUCAUUAAAUUUGC

ENERGY = -20.3

total reads	mature	loop	star
41	40	0	1

Bases 1 to 54

```
           10        20
U---|     U   AUUAA        G
    GAACUC GUC     AUUUGCUA U
    CUUGAG CAG     UAAACGAU C
AGCG^     -   GUGUG        G
   50         40        30
```

NA

---

egr-new-38

hairpin:
GAAAUGCCGAGGGCUUGGAGGACAUCGUAUCUGCAUCAAGGCUCACGGCAUUUCCU
mature:
GAAAUGCCGAGGGCUUGGAGG

ENERGY = -22.9

total reads	mature	loop	star
27	24	0	3

Bases 1 to 56

```
          10        20
--|        A      G  GGA  UCG
  GAAAUGCCG GGGCUU GA   CA   \
  CUUUACGGC CUCGGA CU   GU   U
UC^        A      A  AC-  CUA
     50        40         30
```

NA

---

egr-new-41

hairpin:
AGCAGGGACUGGUUUGAAACUGCCUGCAGUACAAACUCUGCCCCUGCUGC
mature:
UACAAACUCUGCCCCUGCUGC

ENERGY = -22.2

total reads	mature	loop	star
16	14	0	2

Bases 1 to 50

```
          10         20
--       ACU-|     AA    C
  AGCAGGG    GGUUUG  ACUG C
  UCGUCCC    UCAAAC  UGAC U
CG       CGUC^     A-    G
.        40         30
```

NA

---

egr-new-42

hairpin:
GACUGGGUUGCAGGAAAGCGUGAAUAAGUGCUACAUAGAGAACUUAGUCACGCAUACCCAAAGCCCAGUUCA
mature:
CAUACCCAAAGCCCAGUUCA

ENERGY = -30.2

total reads	mature	loop	star
14	7	1	6

Bases 1 to 72

```
          10        20        30
--|        GCA  AAA      A     G  AC
  GACUGGGUU   GG   GCGUGA UAAGU CU  A
  UUGACCCGA   CC   CGCACU AUUCA GA  U
AC^        AAC  AUA      G     A  GA
 70        60        50        40
```

NA

---

egr-new-43

hairpin:
GUCCACGCAGCACAGCGGUCACGAAUGCUGGGCCGAUGAAUGCCACGUGGUCAC
mature:
GUCCACGCAGCACAGCGGUCA

ENERGY = -20.1

total reads	mature	loop	star
21	19	0	2

Bases 1 to 54

```
          10          20
GU--     CA   --| G     A  AA
    CCACG  GCA  CA CGGUC CG  \
    GGUGC  CGU  GU GCCGG GU  U
CACU     AC   AA^ A     -  CG
   50        40         30
```

NA

---

egr-new-45

hairpin:
AAAGGUCAGGAUGGCGGAGUGAAGGGCGGCACUGACGCGUCAUUCCCACCCUCCCCUCCUCCCCUGCCCUUUUC
mature:
AAAGGUCAGGAUGGCGGAGUG

ENERGY = -30.7

total reads	mature	loop	star
8	7	0	1

Bases 1 to 74

```
          10        20        30
--|    U    AU  C    UGA    C  CAC   C
  AAAGG CAGG  GG GGAG   AGGG GG   UGA G
  UUUCC GUCC  CC CCUC   UCCC CC   ACU C
CU^    C    --  U    CCC    A  CUU   G
   70          60        50        40
```

NA

---

egr-new-46

hairpin:
UGAAUGGCUAGAGGCAUCAAGGACAUCCAUACAUACGGUUGCCCUUGAACCGUUUCUACCAUUCAUA
mature:
UGAAUGGCUAGAGGCAUCAAG

ENERGY = -27.1

total reads	mature	loop	star
11	10	0	1

Bases 1 to 67

```
          10          20        30
--       C       A--|     A   U   AUA
  UGAAUGG UAGAGGC   UCAAGG CA CC   C
  ACUUACC AUCUUUG   AGUUCC GU GG   A
AU       -       CCA^     C   U   CAU
      60          50        40
```

NA

---

egr-new-47

hairpin:
UAGUCAGUCAGUCUGUUUGCCUGUAUGGCUGGCUGGCUGACUGGCUG
mature:
UAGUCAGUCAGUCUGUUUGC

ENERGY = -28.6

total reads	mature	loop	star
10	5	0	5

Bases 1 to 47

```
        10        20
|            U  UU   UG
UAGUCAGUCAGUC GU  GCC  \
GUCGGUCAGUCGG CG  CGG  U
^            U  GU   UA
      40        30
```

NA

---

egr-new-48

hairpin:
UGGUGCUUGAUCAUACCACUGAUAGACAUGGAUCAGUUGUAUCAUCAAUGCAUCACA
mature:
UGGUGCUUGAUCAUACCACUGA

ENERGY = -22

total reads	mature	loop	star
88	88	0	0

Bases 1 to 57

```
           10        20
--      -|    C    C      AGA
  UGGUGC UUGAU AUAC ACUGAU   C
  ACUACG AACUA UAUG UGACUA   A
AC      U^    C    U      GGU
      50        40        30
```

NA

---

egr-new-50

hairpin:
GACUUGGAUGGUUCUGCCUAACCAGGGGUGGAGGCCAUCCAAGUAGC
mature:
GUGGAGGCCAUCCAAGUAGC

ENERGY = -26.8

total reads	mature	loop	star
19	19	0	0

Bases 1 to 47

```
          10         20
G--          -|  UG   AA
   ACUUGGAUGG UUC  CCU  C
   UGAACCUACC GAG  GGG  C
CGA          G^  GU   GA
      40        30
```

NA

---

egr-new-51

hairpin:
UAGAGCUUGACGUGUUGGAGACGCGGCUACUCGUGGAUGCUCCAGAGAGGAUGAAGUCACCAAUACGUUGCCAGCUCCAUC
mature:
UAGAGCUUGACGUGUUGGAGAC

ENERGY = -32.9

total reads	mature	loop	star
136	136	0	0

Bases 1 to 81

```
            10        20         30
UA--     --           A   GCGG-| A   G   AU
    GAGCU  UGACGUGUUGG GAC     CU CUC UGG  \
    CUCGA  GUUGCAUAACC CUG     GG GAG ACC  G
CUAC     CC           A   AAGUA^ A   -   UC
80        70        60        50         40
```

NA

---

egr-new-52

hairpin:
ACUAGGAGAGGGGAUGAGGAAGCCUCUUCCUCUCUCCUGGUC
mature:
ACUAGGAGAGGGGAUGAGGA

ENERGY = -29.6

total reads	mature	loop	star
9	8	0	1

Bases 1 to 42

```
         10        20
-|             U    A
 ACUAGGAGAGGGGA GAGG \
 UGGUCCUCUCUCCU CUCC A
C^             U    G
 40        30
```

NA

---

egr-new-55

hairpin:
CGUGGAGGAGUUCGGGAUAGCCCUUUCGUGUGAGUGUUCCGUCUCCUCCACAUC
mature:
CGUGGAGGAGUUCGGGAUAGC

ENERGY = -26.7

total reads	mature	loop	star
21	21	0	0

Bases 1 to 54

```
          10        20
C--|        UU       GCC   UC
   GUGGAGGAG  CGGGAUA   CUU  G
   CACCUCCUC  GCCUUGU   GAG  U
CUA^        U-       ---   UG
   50         40           30
```

NA

---

egr-new-56

hairpin:
GAGUAAUUUCUAUCCGGUUGGUGUUUUCCAGGGAACACUAGUGUGUCCUCUAUGUCCAAGGCAUCACCGGGUAGUUAUUACGC
mature:
UCACCGGGUAGUUAUUACGC

ENERGY = -34.2

total reads	mature	loop	star
64	49	0	15

Bases 1 to 83

```
        10        20            30        40
GA     UU        U         CC----|   A    U
  GUAAU  CUAUCCGG UGGUGUUUU      AGGG ACAC A
  CAUUA  GAUGGGCC ACUACGGAA      UCUC UGUG G
CG     UU        -         CCUGUA^   C    U
  80        70         60        50
```

NA

---

egr-new-57

hairpin:
AAGAGGAGAGGGAGAAGAGAGUUAGCUGUCCAAAGCUUUGCUUACCCCUUCUCCUCCCCCUCUUCU
mature:
AAGAGGAGAGGGAGAAGAGAGUU

ENERGY = -27.5

total reads	mature	loop	star
47	46	0	1

Bases 1 to 65

```
        10              20          30
A       A     A   ------    U-|   GU
 AGAGGAG GGGAG AGA      GAGU  AGCU  C
 UCUUCUC CCCUC UCU      UUCG  UCGA  C
-       -     C   UCCCCA    UU^   AA
     60          50         40
```

NA

---

egr-new-58

hairpin:
AAUGGCACUGGGAUGGAUGGUAGAGUGGAUUCCUACCAUCCUCGUAGUGUGGUUCA
mature:
AAUGGCACUGGGAUGGAUGGUAG

ENERGY = -27.3

total reads	mature	loop	star
33	33	0	0

Bases 1 to 56

```
          10         20
--|  G      G  U         AGUG
  AAU GCACUG GA GGAUGGUAG    \
  UUG UGUGAU CU CCUACCAUC    G
AC^  G      G  -         CUUA
     50          40         30
```

NA

---

egr-new-59

hairpin:
GUGAGAAUACUGGACUAGGAUGCUAUAAUCCAAUGUCAAGUGUUCUCACAA
mature:
GUGAGAAUACUGGACUAGGAUG

ENERGY = -25.2

total reads	mature	loop	star
27	27	0	0

Bases 1 to 51

```
          10         20
--           G   UA-|   GC
  GUGAGAAUACU GAC   GGAU  U
  CACUCUUGUGA CUG   CCUA  A
AA           A   UAA^   AU
50        40         30
```

NA

---

egr-new-60

hairpin:
AGCAACACCUGCGUCUCACAUAAGGUUGUGGUGGCGGUGGUGUUGAUGA
mature:
GUGGUGGCGGUGGUGUUGAUGA

ENERGY = -21.2

total reads	mature	loop	star
51	51	0	0

Bases 1 to 49

```
          10         20
AG--       UG    -|    UA
    CAACACC  CGUC UCACA  A
    GUUGUGG  GCGG GGUGU  G
AGUA       UG    U^    UG
        40         30
```

NA

---

egr-new-61

hairpin:
UGGCGCUUGGUUAUUCUACUGAUAGCCAAAAGUCAGUCGUAUAAUCAACGUGCCGCA
mature:
UGGCGCUUGGUUAUUCUACUGA

ENERGY = -22.7

total reads	mature	loop	star
3524	3524	0	0

Bases 1 to 57

```
           10        20
U--     -|       UCU      AGC
   GGCGC UUGGUUAU   ACUGAU   C
   CCGUG AACUAAUA   UGACUG   A
ACG     C^       UGC      AAA
      50        40        30
```

NA

---

egr-new-62

hairpin:
GUGAUCUGCCAAUUUGCUCUCGGCCGUCGCUCUAAAAUUGGCGGGUCAAAG
mature:
GUGAUCUGCCAAUUUGCUCUC

ENERGY = -20.6

total reads	mature	loop	star
27	27	0	0

Bases 1 to 51

```
          10        20
G--|             GCUCUC   C
   UGAUCUGCCAAUUU      GGC G
   ACUGGGCGGUUAAA      UCG U
GAA^             AUC---   C
50        40            30
```

NA

---

egr-new-63

hairpin:
ACGGUUCGAACAGAAGUUGGCAGAAAGCUUUUCCUCCUUCUGUUCGACCAAUU
mature:
CCUCCUUCUGUUCGACCAAUU

ENERGY = -22.5

total reads	mature	loop	star
28	28	0	0

Bases 1 to 53

```
          10        20
AC--|  U          UU  C   A
    GGU CGAACAGAAG  GG AGA A
    CCA GCUUGUCUUC  CC UUU G
UUAA^  -          CU  U   C
  50         40        30
```

NA

---

egr-new-64

hairpin:
GCCUGAUUUGAGUGCAGCCUCAUGUAGGCAGGACAUCGAAUCAGGAAA
mature:
GCAGGACAUCGAAUCAGGAAA

ENERGY = -21.9

total reads	mature	loop	star
23	23	0	0

Bases 1 to 48

```
          10         20
G--          -| GCA    CA
   CCUGAUUUGA GU   GCCU  \
   GGACUAAGCU CA   CGGA  U
AAA          A^ GGA    UG
       40        30
```

NA

---

egr-new-65

hairpin:
UACGUGCAUUUCAGCUCAUCUUCUCCACUGCUUGGAGUCUGAGGUGAAAAUGCGCGAAUU
mature:
UCUGAGGUGAAAAUGCGCGAAUU

ENERGY = -26.1

total reads	mature	loop	star
93	93	0	0

Bases 1 to 60

```
           10        20
UA--      -|    G    UCUU     CU
    CGUGCA UUUCA CUCA    CUCCA  \
    GCGCGU AAAGU GAGU    GAGGU  G
UUAA      A^    G    CU--     UC
.        50        40
```

NA

---

egr-new-66

hairpin:
CAUCAAUAUUGGCUGUGACAUCGCAACCCGUGUCUACAGGCAAUGUUGACGACG
mature:
CAUCAAUAUUGGCUGUGACAU

ENERGY = -21.6

total reads	mature	loop	star
18	13	0	5

Bases 1 to 54

```
           10         20
CA---         G    -|    CGC
     UCAAUAUUG CUGU GACAU   A
     AGUUGUAAC GACA CUGUG   A
GCAGC         G    U^    CCC
   50        40         30
```

NA

---

egr-new-68

hairpin:
CCAAACCGUUCCAAUAGUCGACAGUUUAAACCCACCGACUACUGGAACGGUUUAAC
mature:
CGACUACUGGAACGGUUUAAC

ENERGY = -24.4

total reads	mature	loop	star
7	6	0	1

Bases 1 to 56

```
         10        20
CC-|          A      ACAGUUU
   AAACCGUUCCA UAGUCG       \
   UUUGGCAAGGU AUCAGC       A
CAA^          C      CACCCAA
     50        40        30
```

NA

---

egr-new-70

hairpin:
CCUCCUUCUGUUCGACCAAUUCAGUCAUGCACCCUCUAACCACUGCGAUUGGUCCAAUAAAGAAGGUGGAG
mature:
CCUCCUUCUGUUCGACCAAUU

ENERGY = -23.4

total reads	mature	loop	star
23	23	0	0

Bases 1 to 71

```
            10        20         30
--  U      --   C        -|   CAUGCAC
  CC CCUUCU  GUU GACCAAUU CAGU       C
  GG GGAAGA  UAA CUGGUUAG GUCA       C
GA  U      AA   C        C^   CCAAUCU
70        60        50        40
```

NA

---

egr-new-71

hairpin:
UUGAAGGUACUGUGAAUUUUGUAUGAGUCCAAACUGGUACAAGAUGCCCAUUCGCGCGGUCCAUG
mature:
UUGAAGGUACUGUGAAUUUUG

ENERGY = -19.6

total reads	mature	loop	star
12	12	0	0

Bases 1 to 65

```
        10                 20         30
UUGAA   U      A--------|         G   C
     GG ACUGUG         AUUUUGUAU AGU C
     CC UGGCGC         UAGAACAUG UCA A
GUA--   -      GCUUACCCG^         G   A
       60         50         40
```

NA

---

egr-new-73

hairpin:
UGGGCUAAAGUUGGUACAUGUACCUAGACGGUUGUGUGCAUGUCUGCUAUGGCCCAUC
mature:
UGGGCUAAAGUUGGUACAUGUA

ENERGY = -23.2

total reads	mature	loop	star
67	67	0	0

Bases 1 to 58

```
          10         20
--|      A   U  U        C   A
  UGGGCUA AGU GG ACAUGUAC UAG C
  ACCCGGU UCG UC UGUACGUG GUU G
CU^      A   -  -        U   G
       50          40         30
```

NA

---

egr-new-74

hairpin:
GGUGCCAUUAGUGUGAUAGGCUCAGCGUUGAUCUAUUCAACAUCAGCCUUCAUGCGCUUGGAGCGU
mature:
GGUGCCAUUAGUGUGAUAGG

ENERGY = -23.7

total reads	mature	loop	star
327	327	0	0

Bases 1 to 66

```
         10        20        30
G-| G   UUA      U     CAGC     UC
  GU CCA   GUGUGA AGGCU    GUUGA  \
  CG GGU   CGUACU UCCGA    CAACU  U
UG^ A   UCG      -     CUA-     UA
     60        50          40
```

NA

---

egr-new-75

hairpin:
AUGGAGGAGAAGGGAAGGAAGAUGUUGUUAAAUCUUCAUCCUCAUCCUCCACAC
mature:
AUGGAGGAGAAGGGAAGGAAGA

ENERGY = -24.1

total reads	mature	loop	star
34	32	0	2

Bases 1 to 54

```
          10        20
A--|      GAA    AG      GUU
   UGGAGGA   GGGA  GAAGAU   G
   ACCUCCU   UCCU  CUUCUA   U
CAC^      AC-    A-      AAU
   50         40          30
```

NA

---

egr-new-76

hairpin:
UGCCUACUUCCUCAGCAAAAUUGGGAGAAAGUAAUGCGAUGUUGUCGUCGUGGCCACAUUCGCCAUAUUGUUGUAGAAGGCGGAAA
mature:
AUAUUGUUGUAGAAGGCGGAAA

ENERGY = -27.1

total reads	mature	loop	star
70	70	0	0

Bases 1 to 86

```
         10        20        30         40
UG-| UA    CU      AAU   GA   A  AA-      U
   CC  CUUC  CAGCAA   UGG  GAA GU   UGCGAUG U
   GG  GAAG  GUUGUU   ACC  CUU CA   GUGCUGC G
AAA^ CG    AU      AU-   G-   A  CCG      U
     80        70          60        50
```

NA

---

egr-new-78

hairpin:
AGGGCUAAAGUACAAGAGAGUGGUAACAGCUGCUGUACUUUAGCCCUCC
mature:
AGGGCUAAAGUACAAGAGAG

ENERGY = -24.4

total reads	mature	loop	star
14	14	0	0

Bases 1 to 49

```
          10        20
--|             AG  AG  G
  AGGGCUAAAGUACA  AG  UG U
  UCCCGAUUUCAUGU  UC  AC A
CC^             CG  G-  A
        40        30
```

NA

---

egr-new-79

hairpin:
CCCGCACUAUGAUUGCCAAACUCCACAGCUCGCAGAGAGGAAGCAUCAAGAGUUGGCAACGGUAGUGCGAUAC
mature:
GUUGGCAACGGUAGUGCGAUAC

ENERGY = -27.2

total reads	mature	loop	star
44	44	0	0

Bases 1 to 73

```
          10        20          30
CC--        GA      A     CACA-   -| GCA
    CGCACUAU  UUGCC AACUC     GC UC   G
    GCGUGAUG  AACGG UUGAG     CG AG   A
CAUA        GC      -     AACUA  A^ GAG
  70        60         50        40
```

NA

---

egr-new-80

hairpin:
CCUCUCUAUCCCUUUUCACGUUAGUAUUGUAUGCUCUACCAGUAUGCUGUGACUAUACGGAUUUUGGCGGAGUUGGGGAGGCA
mature:
CCUCUCUAUCCCUUUUCACGUUA

ENERGY = -27.7

total reads	mature	loop	star
288	288	0	0

Bases 1 to 83

```
            10           20          30
--        --   -  UU--  A   -    AUU-|      CU
  CCUCUCUA  UCC CU    UC CGU UAGU    GUAUGCU  \
  GGAGGGGU  AGG GG    AG GCA AUCA    CGUAUGA  A
AC        UG   C  UUUU  -   U    GUGU^      CC
  80        70        60          50        40
```

NA

---

egr-new-82

hairpin:
ACCACAAAAAACUGCCGAAAACGAUAAGUAAAAUCCUUUUUUGGUAGUUUUUUGUAGGAUU
mature:
UUUGGUAGUUUUUUGUAGGAUU

ENERGY = -23.6

total reads	mature	loop	star
67	67	0	0

Bases 1 to 61

```
           10        20
A--  -                  C-|  AAG
   CC ACAAAAAACUGCCGAAAA  GAU   \
   GG UGUUUUUUGAUGGUUUUU  CUA   U
UUA  A                  UC^  AAA
60        50        40        30
```

NA

---

egr-new-87

hairpin:
AUUUGCUCUGGGUUAGUAGAAGGCAUUUUAAAUGCACUACUAAACCAAGAGACAAAUUGA
mature:
AUUUGCUCUGGGUUAGUAGAA

ENERGY = -21

total reads	mature	loop	star
5555	3992	10	1553

Bases 1 to 60

```
            10         20
---     -    G  -|      AAG     U
   AUUUG CUCU GG UUAGUAG   GCAUU \
   UAAAC GAGA CC AAUCAUC   CGUAA U
AGU     A    A  A^      A--     A
.         50        40          30
```

NA

---

egr-new-91

hairpin:
CUGAUUCGAAACCUCGGCCUUUUUGUUACUGUUCUUUGGGUUUCCCUAACAUGACUCCCACUGCGUAAUUAGAGGAUUGUUUCGGGUGGGC
mature:
CUGAUUCGAAACCUCGGCC

ENERGY = -26.2

total reads	mature	loop	star
56	56	0	0

Bases 1 to 91

```
         10        20        30        40
-|  G         CUCGG     UU      U  UCUU    UU  CCU
 CU AUUCGAAAC      CCUUU  GUUAC GU    UGGG  UC    A
 GG UGGGCUUUG      GGAGA  UAAUG CG    ACCC  AG    A
Cˆ  G         UUA--     U-      -  UC--    UC  UAC
90        80          70            60        50
```

NA

---

egr-new-92

hairpin:
UGUGGGCAAAGUGAGUGGGAUCAAACAUUAUGUCAACAUAUGAAACAUUGGGAAAUCUCAUGCCUUUGCCCACGAA
mature:
UGUGGGCAAAGUGAGUGGGAUC

ENERGY = -28.3

total reads	mature	loop	star
28	28	0	0

Bases 1 to 76

```
          10        20        30
--|          UGA       CAAA  UUAUG   AC
  UGUGGGCAAAG   GUGGGAU    CA     UCA  \
  GCACCCGUUUC   UACUCUA    GU     AGU  A
AAˆ          CG-       AAGG  UACAA   AU
     70          60        50        40
```

NA

---

egr-new-95

hairpin:
CCUCCUUCCUGCUUUUUUUCACCUCGGUGCUGUCCAGCUAACUUGUAGGAAAGUGAAGGAGGCA
mature:
CCUCCUUCCUGCUUUUUUUC

ENERGY = -20.9

total reads	mature	loop	star
18	18	0	0

Bases 1 to 64

```
          10        20          30
--|       CU        U  CCUC   --   G
  CCUCCUUC  GCUUUUUU CA    GGU  GCU U
  GGAGGAAG  UGAAAGGA GU    UCA  CGA C
AC^       --        U  ----   AU   C
   60          50            40
```

NA

---

egr-new-96

hairpin:
AUGCGGAUGGUUAAGGAAUGGGGAGAAUGACCUUGACAGGAGAGUCCCUGAGGUGCCAGUCCCCAGAUCAAACCACCGCAGAC
mature:
AUGCGGAUGGUUAAGGAAUGGG

ENERGY = -34.3

total reads	mature	loop	star
10	10	0	0

Bases 1 to 83

```
          10        20        30        40
A--|    A     AA  AA      GAAUG    UGA    AG
   UGCGG UGGUU  GG  UGGGGA     ACCU   CAGG  A
   ACGCC ACCAA  CU  ACCCCU     UGGA   GUCC  G
CAG^    -     A-  AG      GACCG    ---    CU
  80         70         60            50
```

NA

---

egr-new-97

hairpin:
GGAGGGUAUUAACCGAAGGCACAGAUUUGCCUUCGGCCCCACCCUUCCU
mature:
GGAGGGUAUUAACCGAAGGC

ENERGY = -25.6

total reads	mature	loop	star
10	10	0	0

Bases 1 to 49

```
          10        20
--|      AUUAA         CA
  GGAGGGU     CCGAAGGCA  G
  CUUCCCA     GGCUUCCGU  A
UC^      CCCC-         UU
        40         30
```

NA

---

egr-new-98

hairpin:
GAGAUGAGCAUCCAGUGCUUAGUGAUUGUGUAAGCACUGCUGCUCAUACCACAAUCAUUCAUC
mature:
GAGAUGAGCAUCCAGUGCUUA

ENERGY = -21.4

total reads	mature	loop	star
11	10	0	1

Bases 1 to 63

```
                     10        20
GAG-------------|      UC          GUGA
                AUGAGCA  CAGUGCUUA    \
                UACUCGU  GUCACGAAU    U
CUACUUACUAACACCA^      C-          GUGU
  60        50        40          30
```

NA

---

egr-new-99

hairpin:
UGCAUUUCAUCACCUACUCCCAUCACUUACCGUGGUGGUGACACUUUCGGUGUGAUGGAUUGAAUAAGAGUGCACA
mature:
AUGGAUUGAAUAAGAGUGCACA

ENERGY = -27.4

total reads	mature	loop	star
8	8	0	0

Bases 1 to 76

```
            10        20        30
--       CA--|  CCUACUC       UU    U-    G
  UGCAUUU    UCA       CCAUCAC  ACCG  GGUG U
  ACGUGAG    AGU       GGUAGUG  UGGC  UCAC G
AC       AAUA^  UA-----       --    UU    A
     70        60                50        40
```

NA

---

egr-new-100

hairpin:
GUAGAUCGUUUGGCGAUAAGCGGUGAACAGUGAUUUACAGUGAAGGCCAAACAUCUACCA
mature:
GUAGAUCGUUUGGCGAUAAGC

ENERGY = -20.7

total reads	mature	loop	star
40	40	0	0

Bases 1 to 60

```
          10        20        30
--|      C       GAUAA  G     CA
  GUAGAU GUUUGGC     GC GUGAA  G
  CAUCUA CAAACCG     UG CAUUU  U
AC^      -       GAAG-  A     AG
.         50         40
```

NA

---

egr-new-104

hairpin:
CGUGGGGCUUGUGGUACUUCUGGGUGCGGGUGCAGUGCGUGUGUUGGCGCAGGGGUAAGCCAGUCCCUAUUUC
mature:
CGUGGGGCUUGUGGUACUUCUG

ENERGY = -28.8

total reads	mature	loop	star
7	7	0	0

Bases 1 to 73

```
           10        20        30
CGU---|     U  GG       GG    G  UG  GU
      GGGGCU GU  UACUUCU  GUGC GG  CA  \
      CCCUGA CG  AUGGGGA  CGCG UU  GU  G
CUUUAU^     C  A-       --    G  GU  GC
  70        60            50        40
```

NA

---

egr-new-106

hairpin:
GAGCGGCUGUUGAACUAGUACCGACCUUUGAUAAUUACCCAACAGACAGUUCGUGUAUUCAAGUUCAAAUAGCCGCAUUUG
mature:
GAGCGGCUGUUGAACUAGUACC

ENERGY = -26

total reads	mature	loop	star
8	8	0	0

Bases 1 to 81

```
            10          20          30
GA---       -       --     -   C  --|  AUAAU
     GCGGCUG UUGAACU  AGUAC CGA CU  UUG     U
     CGCCGAU AACUUGA  UUAUG GCU GA  GAC     A
GUUUA       A       AC     U   U  CA^  AACCC
80        70        60        50        40
```

NA

---

egr-new-107

hairpin:
AGAAGUUGUUAGCUGGUAGGUAUGAUUAGCUACCAUCUCUCAGCUUCAUU
mature:
AGAAGUUGUUAGCUGGUAGG

ENERGY = -21.5

total reads	mature	loop	star
17	17	0	0

Bases 1 to 50

```
          10        20
A--|      UU  C      G  U
   GAAGUUG  AG UGGUAG UA G
   CUUCGAC  UC ACCAUC AU A
UUA^      UC  U      G  U
.        40        30
```

NA

---

egr-new-108

hairpin:
AACUUAAAUGGUCGUGUGGCUGCAUUACGCCGCACUGCCAUUUGAUGUCAC
mature:
AACUUAAAUGGUCGUGUGGC

ENERGY = -23.1

total reads	mature	loop	star
10	10	0	0

Bases 1 to 51

```
           10        20
A--  -|       C       UGC
   AC UUAAAUGGU GUGUGGC   A
   UG AGUUUACCG CACGCCG   U
CAC  U^       U       CAU
50        40        30
```

NA

---

egr-new-109

hairpin:
CGGACAGUGCACGGGUAUUUUGCUUUGCCUGUGCGUCACGCGCCGGGGGCAGGGGCAGGGCGGCGGUGCCCACAUUGGACGAC
mature:
CGGACAGUGCACGGGUAUUUU

ENERGY = -42.3

total reads	mature	loop	star
6	6	0	0

Bases 1 to 83

```
          10         20        30        40
--| GA     CAC       -           G   G  A  C
  CG  CAGUG   GGGUAUU UUGCUUUGCCU UGC UC CG \
  GC  GUUAC   CCCGUGG GGCGGGACGGG ACG GG GC G
CA^ AG     A--       C           G   G  -  C
  80          70        60        50
```

NA

---

egr-new-113

hairpin:
CGGUGUGGGAGUAGAUUUCUGAGAACAUUUUAGUGACCUUAGAGGAUAUGCUCAGCACUGUU
mature:
CGGUGUGGGAGUAGAUUUCUG

ENERGY = -22.4

total reads	mature	loop	star
7	7	0	0

Bases 1 to 62

```
          10         20        30
--|      GG     GA-        AA   U
  CGGUGU  GAGUA   UUUCUGAG  CAU U
  GUCACG  CUCGU   GGAGAUUC  GUG U
UU^      A-     AUA        CA   A
 60          50        40
```

NA

---

egr-new-114

hairpin:
GUGCACACGUAUGUGUAUGCAUCUAUGCGCGGGCCUACACACACGUGCGCACGA
mature:
GUGCACACGUAUGUGUAUG

ENERGY = -24.5

total reads	mature	loop	star
6	6	0	0

Bases 1 to 54

```
          10        20
--|   A     A      U  A  UAU
  GUGC CACGU UGUGUA GC UC   \
  CACG GUGCA ACACAU CG GG   G
AG^   C     C      C  -  CGC
   50        40         30
```

NA

---

egr-new-115

hairpin:
ACUGAGGGCGGUAGUGUUUUUUUCGUUGACCAUCAAUGAAAUUGGAUGUGGUUGGGAAUGAGAGACACUCACCGCUUUUUAGAGC
mature:
ACUGAGGGCGGUAGUGUUU

ENERGY = -30.9

total reads	mature	loop	star
6	6	0	0

Bases 1 to 85

```
           10         20          30
A--  -         -          --  G UG     ---|   G
   CU GAGGGCGGU AGUGUUUUUU  UC U  ACCAU   CAAU \
   GA UUUUCGCCA UCACAGAGAG  AG G  UGGUG   GUUA A
CGA  U         C          UA  - GU     UAG^   A
    80        70        60         50        40
```

NA

---

egr-new-118

hairpin:
ACGUUCGCGCACGUCCAGCGGAGAUCCUGCCAUCACGGUGUUAGGACUCUUCUGACAUGCAUGAACGAGU
mature:
UCUGACAUGCAUGAACGAGU

ENERGY = -30.4

total reads	mature	loop	star
62	62	0	0

Bases 1 to 70

```
          10        20        30
A--|     C   C   C  C    A     C   C
   CGUUCG GCA GUC AG GGAG UCCUG CAU A
   GCAAGU CGU CAG UC UCUC AGGAU GUG C
UGA^     A   A   -  U    -     U   G
.        60         50         40
```

NA

---

egr-new-119

hairpin:
GGGAGGGAGUGAGGGAGGUAGAUAGUGUCCCACUGCCUUCACAUCCUCCGUA
mature:
CCACUGCCUUCACAUCCUCCGUA

ENERGY = -24.3

total reads	mature	loop	star
5	5	0	0

Bases 1 to 52

```
          10        20
G--     GA       A   A-|  A
   GGAGG  GUGAGGG GGU  GAU \
   CCUCC  CACUUCC UCA  CUG G
AUG     UA       G   CC^  U
 50        40        30
```

NA

---

egr-new-120

hairpin:
UUUCAUCACCCUACCAGAUUGCUCUUAUUCUUUAGUUGGAGCUUAAGCAUCAGCUUUGGAGGUACCCUCAUGGGUGUGAAUUA
mature:
UUUCAUCACCCUACCAGAUUGCUCU

ENERGY = -24.2

total reads	mature	loop	star
6	6	0	0

Bases 1 to 83

```
          10        20        30        40
U--|   U     UACC  AU    CUUAU   UU     GA  U
   UUCA CACCC    AG  UGCU     UCU  AGUUG  GC U
   AAGU GUGGG    UC  AUGG     AGG  UCGAC  CG A
AUU^   -     UAC-  CC    -----   UU     UA  A
  80         70              60        50
```

NA

---

egr-new-122

hairpin:
GGUGGUGGUGGUGGUGGCGGUGAAGAUGACGUGUGUUUCUGAGUCUGCUUUAUCAUCACCAUCGUCA
mature:
CUGCUUUAUCAUCACCAUCGUCA

ENERGY = -28.5

total reads	mature	loop	star
401	370	3	28

Bases 1 to 67

```
         10          20          30
- GU            --      UGA-|  U   G
 G  GGUGGUGGUGGU  GGCGG    AGA GAC U
 C  CUACCACUACUA  UCGUC    UCU UUG G
A UG            UU      UGAG^  -   U
      60         50         40
```

NA

---

egr-new-123

hairpin:
GGGGAGGCGACGCUAGGGAAUAAGUAAUUAGGCUUGUCCCUUUAUUCGUCUUCCUGU
mature:
GUCCCUUUAUUCGUCUUCCUGU

ENERGY = -26.7

total reads	mature	loop	star
5	5	0	0

Bases 1 to 57

```
          10        20
--|         CGCU     A     AA
  GGGGAGGCGA    AGGGA UAAGU  U
  UCCUUCUGCU    UCCCU GUUCG  U
UG^         UAUU     -     GA
      50        40         30
```

NA

---

egr-new-124

hairpin:
AACCGGGUUGGUGGGAGGCAUGGUCAUGCCCCCCACUUUCCCGCCUUU
mature:
AACCGGGUUGGUGGGAGGCAUG

ENERGY = -27.6

total reads	mature	loop	star
5	5	0	0

Bases 1 to 48

```
          10        20
AAC--|   UU      A     G
     CGGG  GGUGGG GGCAU G
     GCCC  UCACCC CCGUA U
UUUCC^   UU      C     C
       40        30
```

NA

---

egr-new-125

hairpin:
CGUCUGCAUGUCUUUGGGGCCCUACAAAGGGUGUCUGCGGGCGGAUGGC
mature:
GGGUGUCUGCGGGCGGAUGGC

ENERGY = -20.9

total reads	mature	loop	star
1854	1854	0	0

Bases 1 to 49

```
          10        20
--|      A   CUUU  G     A
  CGUCUGC UGU    GG GCCCU C
  GUAGGCG GCG    CU UGGGA A
CG^      G   U---  G     A
        40           30
```

NA

---

egr-new-126

hairpin:
CCUCGUCUCGUCUCGUCUCGCCUCGGAAGAGAGAGGAGGGAAGGGAGAGGCG
mature:
CCUCGUCUCGUCUCGUCUC

ENERGY = -26.7

total reads	mature	loop	star
229	229	0	0

Bases 1 to 52

```
          10        20
--|  G    G    GU   GC   G
  CCUC UCUC UCUC  CUC  CUC G
  GGAG AGGG AGGG  GAG  GAG A
GC^  -    A    AG   A-   A
 50         40         30
```

NA

---

egr-new-127

hairpin:
CGGAGGGGUCUGGGAGCUUCUGGCAUGGCCCAAGGGUCAUAAUGCUGGGCAAGAAGUACAAUGACCGUGUCCUUUAAU
mature:
CGGAGGGGUCUGGGAGCUUCUG

ENERGY = -27.6

total reads	mature	loop	star
5	5	0	0

Bases 1 to 78

```
              10        20                30
C-----   GG-    UGGGA       -----    ---|    C
      GGA   GGUC     GCUUCU     GGCA   UGGCC A
      CCU   CCAG     UGAAGA     UCGU   ACUGG A
UAAUUU   GUG    UAACA       ACGGG    AAU^    G
       70        60        50        40
```

NA

---

egr-new-128

hairpin:
AAAUGGACCACAAAGAACGCGCUAAUAGACAGCGCGUCCUUUGUGGUCCAUUUGU
mature:
AAAUGGACCACAAAGAACGC

ENERGY = -35.8

total reads	mature	loop	star
31	31	0	0

Bases 1 to 55

```
          10        20
--|              A       AAU
  AAAUGGACCACAAAG ACGCGCU   \
  UUUACCUGGUGUUUC UGCGCGA   A
UG^              C       CAG
    50        40        30
```

NA

---

egr-new-130

hairpin:
CAUCCUAAUUCCAUGCUUGUUUUCAGACUCUGCCGUCGAUUUGGACGCAUUGUCUGGGGACGAGGAUAGCGAUGGGAUAUC
mature:
CAUCCUAAUUCCAUGCUUGUUUUCA

ENERGY = -33.5

total reads	mature	loop	star
5	5	0	0

Bases 1 to 81

```
          10        20        30        40
C--|     AUUCC  G      UU      UC   C   GA
   AUCCUA     AU CUUGUU  CAGAC  UGC GUC  U
   UAGGGU     UA GAGCAG  GUCUG  ACG CAG  U
CUA^     AGCGA  G      GG      UU   -   GU
80        70        60        50
```

NA

---

egr-new-131

hairpin:
AAGAAGACCAUAGACACGCACUGCCACAGGCGUACCACAUUUCGGUGCGAUAGACAGGUGUGCGCGUAUGCGUUUUCAAGUGC
mature:
AAGAAGACCAUAGACACGCACU

ENERGY = -28.7

total reads	mature	loop	star
9	9	0	0

Bases 1 to 83

```
             10        20        30
AA----      -|  AG  A     U   ACAGG--      ACA
      GAAGAC CAU  AC CGCAC GCC       CGUACC   \
      CUUUUG GUA  UG GCGUG UGG       GCGUGG   U
CGUGAA      C^  --  C     -   ACAGAUA      CUU
  80        70          60         50
```

NA

---

egr-new-132

hairpin:
UGGUGUGGAGCUGUAGAAUAUGGCCGGGAACUCCUGCUAUCAGCAGUAUUUGUUCCCUCUGUGAAAUGUCUGCCCCACAGCAUC
mature:
UGGUGUGGAGCUGUAGAAUAUG

ENERGY = -28.1

total reads	mature	loop	star
5	5	0	0

Bases 1 to 84

```
          10         20        30
--  G     A  UGUAGAA-     CC      UC---|   UA
  UG UGUGG GC        UAUGG  GGGAAC     CUGC  \
  AC ACACC CG        GUGUC  CCCUUG     GACG  U
CU  G     C  UCUGUAAA     U-      UUUAU^   AC
   80        70        60         50
```

NA

---

egr-new-134

hairpin:
GCGUAGGUGCGCGGGGCGAUAUCGCUUGGACAUACUGUACAGUUAAGUGAAUUAUCUCCCUUCCGCAUUUAGCCC
mature:
UCCCUUCCGCAUUUAGCCC

ENERGY = -26.4

total reads	mature	loop	star
47	47	0	0

Bases 1 to 75

```
          10         20          30
--  G        C-    C    --|      GACAU   G
  GC UAGGUGCG  GGGG GAUA  UCGCUUG     ACU U
  CG AUUUACGC  UCCC CUAU  AGUGAAU     UGA A
CC  -        CU    U    UA^      -----   C
     70        60        50              40
```

NA

---

egr-new-141

hairpin:
AGGUACUAGGGCUACUGGCAUGGCCUGAAGGCUAGCCCACCCGGUGUGGUACAAUGGCCGAGCCAAAAGUGGUUCUUUACCUAA
mature:
AGGUACUAGGGCUACUGGCAUG

ENERGY = -36.2

total reads	mature	loop	star
20	20	0	0

Bases 1 to 84

```
          10            20         30         40
--      CU         ---|    A      UGAAG    GC   A
  AGGUA  AGGGCUAC   UGGC UGGCC      GCUA  CC C
  UCCAU  UCUUGGUG   ACCG GCCGG      UGGU  GG C
AA      U-         AAA^    A      UACA    GU   C
   80          70         60         50
```

NA

---

egr-new-143

hairpin:
AGGACACAGAAGGUGGAACGGAGGCAUCGCUAAUCGAGUAGUGACUUUUCUCCUUUUCACUUCGUGUGUGUAUG
mature:
AGGACACAGAAGGUGGAACGGAGGC

ENERGY = -27

total reads	mature	loop	star
18	18	0	0

Bases 1 to 74

```
          10         20            30
AGG--      GA         C      CA--|      AU
     ACACA  AGGUGGAA GGAGG    UCGCUA   C
     UGUGU  UUCACUUU CCUCU    AGUGAU   G
GUAUG      GC         U      UUUC^      GA
   70         60         50         40
```

NA

---

egr-new-145

hairpin:
AGAAUGCCCGCAAUAUUUCUGGCGCCGUAUGUCCUAGCUCCGCUAGGGUUGUUAGCCAGCUGUUGCGGUCAUGUUG
mature:
AGAAUGCCCGCAAUAUUUCUG

ENERGY = -29.8

total reads	mature	loop	star
5	5	0	0

Bases 1 to 76

```
         10        20        30
AGA-|  C        UUU     GCCGUAUG       U
    AUG CCGCAAUA   CUGGC        UCCUAGC \
    UAC GGCGUUGU   GACCG        GGGAUCG C
GUUG^  U        C--     AUUGUU--       C
     70        60          50
```

NA
