# Supplementary figures and images for: Genome-wide sequencing of small RNAs reveals a tissue-specific loss of conserved microRNA families in Echinococcus granulosus
Source: BMC Genomics. 2014 Aug 29;15(1):736. doi: 10.1186/1471-2164-15-736 (PMC4156656; doi:10.1186/1471-2164-15-736)

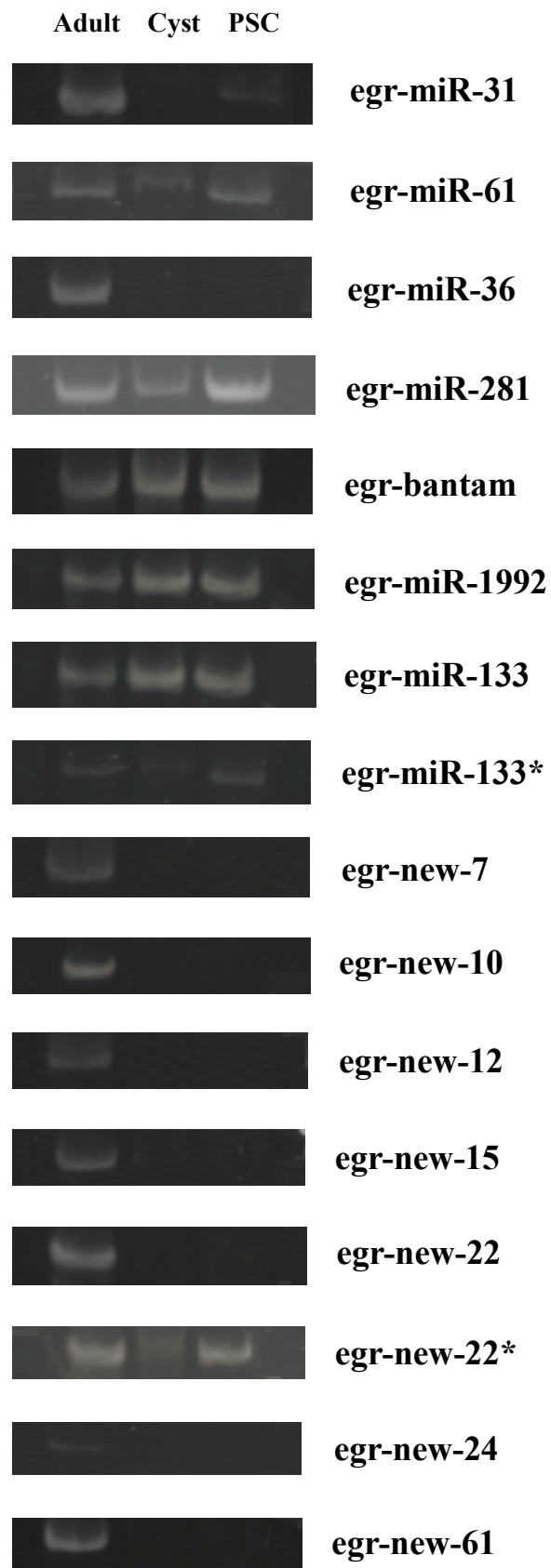

miR-3479   miR-3479\*   new-17\*   new-17   new-28   new-29   marker

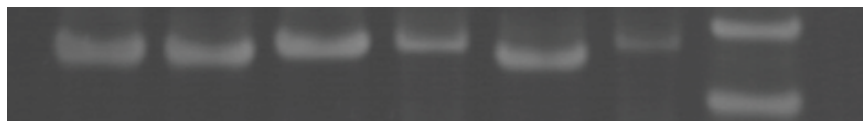

new-51   new-65   new-87\*   new-87   new-125   marker

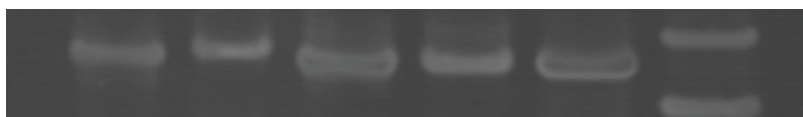

Supplement: Supplementary file 3 — Additional file 3: Figure S1: Confirmation of expression of novel miRNAs using stem-loop PT PCR. (PDF 443 KB) [file 12864_2014_6407_MOESM3_ESM.pdf]

Adult   Cyst   PSC

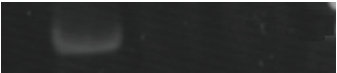

egr-new-15

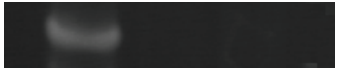

egr-new-22

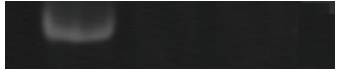

egr-new-61

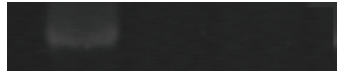

egr-new-12

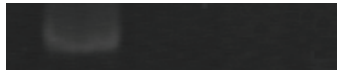

egr-new-7

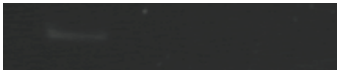

egr-new-24

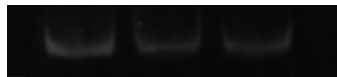

5.8s rRNA

Supplement: Supplementary file 5 — Additional file 5: Figure S3: Stem-loop semi-quantitative PCR of six new E. granulosus miRNAs with 5.8 s rRNA as an internal control. (PDF 294 KB) [file 12864_2014_6407_MOESM5_ESM.pdf]

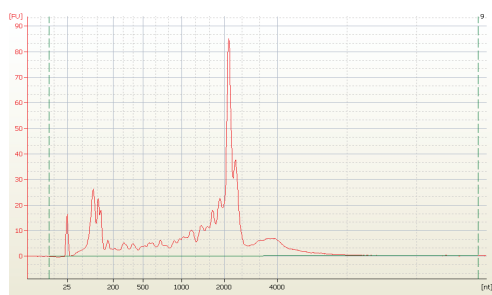

PSC (RIN 7.0)

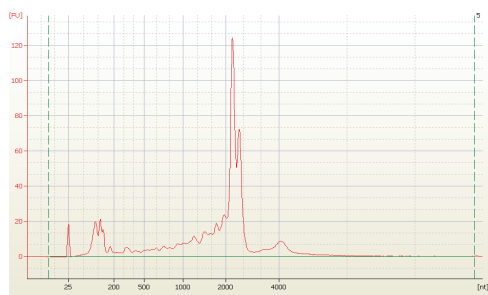

Adult worm (RIN 6.5)

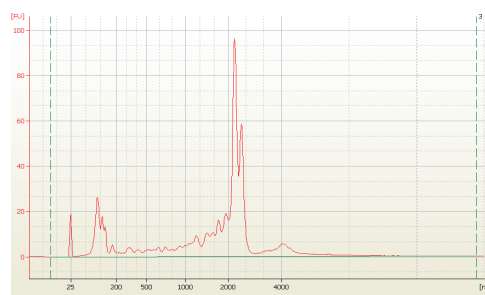

Cyst (RIN 6.8)

Supplement: Supplementary file 6 — Additional file 6: Figure S4: The RNA integrity (RIN) of the Samples of the 3 different life cycle stages. (PDF 298 KB) [file 12864_2014_6407_MOESM6_ESM.pdf]
