# Supplementary material for: Genome-wide sequencing of small RNAs reveals a tissue-specific loss of conserved microRNA families in Echinococcus granulosus
Source: BMC Genomics. 2014 Aug 29;15(1):736. doi: 10.1186/1471-2164-15-736 (PMC4156656; doi:10.1186/1471-2164-15-736)
Supplement: Supplementary file 4 — Additional file 4: Figure S2: Sequence alignment of six new E. granulosus miRNAs in two newly found cluster. (PDF 262 KB) [file 12864_2014_6407_MOESM4_ESM.pdf]

```
             *** ****         ** *  **               * ***        *         *
egr-new-12   UGGCGCUUGA UUUCAACACU GUUAGAAG-- ------CAAC AGUGUUGAUA CCAGGCAUUC ACC
egr-new-24   UGGCGCUUGA CCAUGCUACU GAUAGACG-- ----CAAAUC AGUUGUAUCA UCAAUGCGCC GCA
egr-new-7    UGGCGCUUUC UAACUUUACU GAUAGUGA-- ----UUUAUC AGUAAAGUCA UAAAUGCACC UCA
egr-new-61   UGGCGCUUGG UUAUUCUACU GAUAGCCA-- ----AAAGUC AGUCGUAUAA UCAACGUGCC GCA
egr-new-15   UGGAGCUUGG UGACAAGACU GACAGACA-- ----AGUGGC AGUGUUGCUA UCAAUGCUCC GCA
egr-new-22   UGGCGCUUGA UUUCAACGCU GAUAGAAAAC ACCGUACAUC AGUGUUGAUG UCAGGUUGCU UCU

             *** ****         ** *  **                 ***        *
emu-new-24   UGGCGCUUGA CCAUGCUACU GAUAGACG-- ----CAAAUC AGUUGUAUCA UCAAUGCGCC GCA
emu-new-61   UGGCGCUUGG UUAUUCUACU GAUAGCCA-- ----AAAGUG AGUCGUAUAA UCAACGCGCC GCA
emu-new-7    UGGCGCUUUC UAACUUUACU GAUAGUGA-- ----UUUAUC AGUAAAGUCA UAAAUGCGCC UCA
emu-new-15   UGGAGCUUGG UGACAAGACU GACAGACA-- ----AGUGGC AGUGUUGCUA UCAAUGCUCC GUA
emu-new-12   UGGCGCUUGA UUUCAACACU GUUAGAAG-- ------CAAC AGUGUUGAUA CCAGGCAUUC ACC
emu-new-22   UGGCGCUUGA UUUCAACGCU GAUAGAAAAC ACCGUACAUC AGUGUUGAUG UCAGGUUGCU UCU
```
